# Stimuli-Responsive Properties of Supramolecular Gels Based on Pyridyl-*N*-oxide Amides

**DOI:** 10.3390/gels9020089

**Published:** 2023-01-20

**Authors:** Sreejith Sudhakaran Jayabhavan, Baldur Kristinsson, Dipankar Ghosh, Charlène Breton, Krishna K. Damodaran

**Affiliations:** Department of Chemistry, Science Institute, University of Iceland, Dunhagi 3, 107 Reykjavík, Iceland

**Keywords:** supramolecular gels, isomeric LMWGs, pyridyl *N*-oxide, stimuli-responsive systems, anion/cation-responsive gels, metallogels, cadmium sensor

## Abstract

The nature of functional groups and their relative position and orientation play an important role in tuning the gelation properties of stimuli-responsive supramolecular gels. In this work, we synthesized and characterized mono-/bis-pyridyl-*N*-oxide compounds of *N*-(4-pyridyl)nicotinamide (**L_1_**–**L_3_**). The gelation properties of these *N*-oxide compounds were compared with the reported isomeric counterpart mono-/bis-pyridyl-*N*-oxide compounds of *N*-(4-pyridyl)isonicotinamide. Hydrogels obtained with **L_1_** and **L_3_** were thermally and mechanically more stable than the corresponding isomeric counterparts. The surface morphology of the xerogels of di-*N*-oxides (**L_3_** and **diNO**) obtained from the water was studied using scanning electron microscopy (SEM), which revealed that the relative position of *N*-oxide moieties did not have a prominent effect on the gel morphology. The solid-state structural analysis was performed using single-crystal X-ray diffraction to understand the key mechanism in gel formation. The versatile nature of *N*-oxide moieties makes these gels highly responsive toward an external stimulus, and the stimuli-responsive behavior of the gels in water and aqueous mixtures was studied in the presence of various salts. We studied the effect of various salts on the gelation behavior of the hydrogels, and the results indicated that the salts could induce gelation in **L_1_** and **L_3_** below the minimum gelator concentration of the gelators. The mechanical properties were evaluated by rheological experiments, indicating that the modified compounds displayed enhanced gel strength in most cases. Interestingly, cadmium chloride formed supergelator at a very low concentration (0.7 wt% of **L_3_**), and robust hydrogels were obtained at higher concentrations of **L_3_**. These results show that the relative position of *N*-oxide moieties is crucial for the effective interaction of the gelator with salts/ions resulting in LMWGs with tunable properties.

## 1. Introduction

Soft materials based on stimuli-responsive systems [1,2,3,4] have gained widespread interest because of their tunable properties with respect to external stimuli such as electricity, light, heat, voltage, magnetic field, mechanical stress, pH, and salts/ions. Supramolecular gels based on low-molecular-weight gelators (LMWGs) [5,6,7,8,9,10,11,12,13,14] are an excellent class of stimuli-responsive materials with intriguing potential applications, such as sensors, dynamic gels, tissue engineering, and as media for crystal growth and catalysis [14,15,16,17,18,19,20,21,22,23,24,25,26]. The self-assembly of gelator molecules in a solvent medium leads to the formation of LMWGs with a three-dimensional fibrous network stabilized by various non-covalent interactions. The self-assembly process depends on various parameters [27,28,29,30,31,32,33], such as temperature, pressure, sound, solvent, functional groups, and gelator structure, which can significantly affect the gel network. The nature of the non-bonding interaction and the molecular structure of the gelator play a crucial role in gel network formation. However, predicting the mechanism of the self-assembly process and the gel structure is challenging, because the non-covalent interactions are dynamic in nature. The self-assembly process also depends on the spatial orientation of the functional groups. Understanding the role of building blocks/functional groups and the nature of the non-bonding interactions will help researchers to design smart materials based on LMWGs with intriguing properties. The formation of a one-dimensional (1D) hydrogen-bonded chain can be considered as one of the key primary interactions for the gel network formation in LMWGs [6,8,34]. The incorporation of supramolecular synthons [35] or functional groups with hydrogen-bond functionalities such as urea and amide that can assemble into a 1D array have extensively been used to generate LMWGs, which indicates the importance of the 1D hydrogen-bonded chain [6,8].

LMWGs based on amide groups as supramolecular synthons are an important class of supramolecular gels with tunable properties [36,37,38,39]. Amide-based LMWGs display complementary N–H ··O=C interactions arising from the N−H donor and C=O acceptor of the amide moieties to form a one-dimensional chain, which self-assembles to a three-dimensional network (3D) via cooperative and unidirectional hydrogen bonding [40]. These 3D networks can immobilize solvent molecules to form organo/hydrogels [41,42,43,44,45,46,47]. The adjacent functional groups strongly influence the hydrogen-bonding synthons, and the gelation properties can be altered by introducing moieties that interact with the amide/urea groups. For example, attaching the pyridyl group to the amide moiety can change the intermolecular interaction involving urea/amide groups, which leads to a N···H–N hydrogen-bonding synthon due to the interaction between urea/amide groups and the pyridyl moiety [45,48]. The addition of pyridyl functionality has resulted in highly robust pyridyl amide LMWGs [45,48,49,50], at typical low gelator concentrations, which can be utilized as stimuli-responsive supramolecular systems [11,51,52,53,54]. Incorporating pyridyl amide moieties offers the possibility of synthesizing several isomers, depending on the relative position of the pyridyl nitrogen atom. Furthermore, they provide several advantages, such as ease of obtaining crystalline materials and modification of the pyridyl groups by simple organic reactions. For example, the gelation properties of bis-pyridyl LMWGs can be tuned by replacing the pyridyl group with the corresponding pyridyl-*N*-oxide [55], resulting in pyridyl-*N*-oxide LMWGs.

**Scheme 1:**
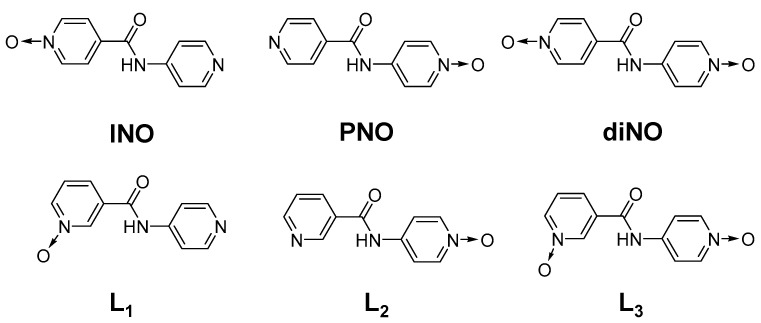
Isomeric mono/di-*N*-oxides of bis-pyridyl amides.

Compounds based on pyridyl-*N*-oxide moieties have gained widespread interest in synthetic chemistry, biochemistry, and pharmacology due to their intriguing potential applications in medicinal science [56,57,58,59]. This is presumably due to the characteristic of the N–O bond [60], which can be considered as a NO donating bond with an important contribution from the oxygen atom (ON back-donation). The substituents play an important role in the stability of the N–O bond; for example, additional stability can be achieved by adding electron-withdrawing substituents, but a reverse trend is observed for electron-donating groups [60]. Incorporating pyridyl-*N*-oxide moieties will lead to enhanced hydrogen bonding and increased solubility in water [55,61] because of the hydrogen-bonding capabilities of the pyridyl-*N*-oxide moiety. Thus, pyridyl-*N*-oxides have a great prospect as hydrogelators, but, surprisingly, the utilization of this functionality in LMWGs is unexplored [55,62,63]. The relative position of the functional groups plays a crucial role in gel network formation in pyridyl-amide/urea gelators, and compounds derived from the *N*-(4-pyridyl) moiety were proved to be superior gelators over the other positional isomers [45,49]. The *N*-oxide moieties could also play an important role in the self-assembly process and the gelation properties of LMWGs [64], and, to the best of our knowledge, the role of the relative position in the gelation properties has not been reported for pyridyl amide *N*-oxides. We have reported the gelation ability of mono-/bis-pyridyl-*N*-oxide compounds of N-(4-pyridyl)isonicotinamide (4PINA) [55] and have shown that bis-pyridyl-*N*-oxide (**diNO**, Scheme 1) displayed better gelation properties compared to mono-pyridyl-*N*-oxides. In this work, we are analyzing the role of the relative position of *N*-oxide moieties in gel network formation by comparing the gelation ability of isomeric pyridyl-*N*-oxide amides in water. The application of the isomeric mono-/bis-pyridyl-*N*-oxide amide LMWGs as sensors will be evaluated by analyzing the stimuli-responsive properties of these LMWGs towards various cations and anions.

## 2. Results and Discussion

We analyzed the role of positional isomers of *N*-oxide moieties in gelation properties by replacing the isonicotinic acid *N*-oxide with nicotinic acid *N*-oxide to form *N*-(4-pyridyl)nicotinamide *N*-oxide compounds (4PNANO, Scheme 1). The mono-pyridyl-*N*-oxide compounds of 4PNANO (**L_1_** and **L_2_**) were synthesized by reacting the corresponding *N*-oxide amine/acid (Schemes S1 and S2), but the bis-pyridyl-*N*-oxide compound was synthesized by oxidizing *N*-(4-pyridyl) nicotinamide with 3-chloroperoxybenzic acid (Scheme S3).

### 2.1. Gelation Experiments

The ability of **L_1_**–**L_3_** to form hydrogels was evaluated in water (1.0 wt%) or aqueous mixtures (1.0 wt/v%). In a typical experiment, 10.0 mg of the compound in 1.0 mL water/aqueous mixtures was heated in a sealed vial to obtain a clear solution, and the mixture was cooled to room temperature. The solution was left undisturbed until gelation was observed, which was confirmed by the vial inversion test. The results indicated that **L_1_**-**L_3_** did not form a gel in water at 1.0 wt%, and the experiments were performed at higher concentrations of the gelators. **L_1_** and **L_3_** formed gels in water at 2.0 wt%, but **L_2_** did not form a gel. The minimum gelator concentration (MGC) required to form the gel network was evaluated by adding different amounts of the gelator (10.0–30.0 mg) to 1.0 mL water. The gelation experiments at various concentrations indicated that the MGC of **L_1_** and **L_3_** was 1.8 wt% in water. We also tested the gelation properties of **L_1_**-**L_3_** in aqueous mixtures (1:1, *v*/*v*) of high polar solvents such as MeOH, EtOH, DMF, and DMSO, and the results were similar to the experiments performed in water (Table 1). Gels were obtained in all cases for **L_1_** and **L_3_** at 2.0 wt/v%, indicating that the presence of cosolvents did not affect the *N*-oxide gel’s self-assembly process.

### 2.2. Thermal Stability

The thermal stabilities of the gels were evaluated by measuring the temperature at which the gel network collapsed, and gels underwent phase transformation to a solution, which is known as gel-to-solution transition temperature (*T_gel_*). A small spherical glass ball was placed on top of the preformed gels at various concentrations of the gelators in the standard vials, and the temperature was increased at a constant rate. As the temperature increased, the ball touched the bottom of the vial, and this temperature was recorded as the *T_gel_*. We performed the *T_gel_* experiments at the MGC of the gelators in water, and the *T_gel_* values for **L_1_** and **L_3_** were 128.0 °C and 76.9 °C, respectively. The experiments performed at higher concentrations of **L_3_** showed an increase in *T_gel_* value (92.9 °C), and the results indicated that increasing the concentration of the gelators enhanced the thermal stability of the gel network. The thermal stabilities of **L_1_** and **L_3_** were analyzed in various aqueous solvents (Table 1), and the *T_gel_* values were lower compared to the corresponding hydrogels. This was presumably due to the favorable interaction between the gelator molecules and the polar solvents, leading to enhanced solubility of the gelators in these mixed solvents, except for **L_3_** in DMSO/water (1:1, *v*/*v*). We compared the MGC value and the thermal stability of **L_3_** with the isomeric *N*-oxides (**diNO**) [55] to see whether the relative position of the *N*-oxide moieties affected the self-assembly process and the gel state properties. The MGC for **L_3_** in water was 1.8 wt%, whereas a higher MGC was observed for isomeric **diNO** (4.0 wt%). The thermal stability of the **L_3_** network in water was also higher than the isomeric **diNO**, and these results indicate that changing the isonicotinic *N*-oxide to nicotinic *N*-oxide leads to better gel network formation.

### 2.3. Rheology

Rheology is an important tool in studying the deformation and flow characteristics of supramolecular gels [65,66]. The mechanical strength of the gelators was analyzed by performing the amplitude- and frequency-sweep experiments on the hydrogels of **L_1_** and **L_3_** at 2.0 wt%. Initially, an oscillatory strain-sweep experiment was performed to evaluate the linear viscoelastic region (LVR), because the gel network undergoes reversible deformation inside LVR. The results demonstrated that **L_1_** and **L_3_** hydrogels had a narrow LVR, as the storage modulus G’ declined after 0.02% of the shear strain (Figure S1). An abrupt decrease in the G’ is observed at the crossover point [65,66], where the gel breaks into a viscous fluid. The crossover points for **L_1_** and **L_3_** hydrogels were within the range of 1.0–5.0% of the shear strain. The frequency-sweep experiments showed constant elastic (G′) and viscous (G′′) moduli with a frequency range of 0.1–10.0 Hz at a constant strain of 0.02% (within LVR).

**Figure 1:**
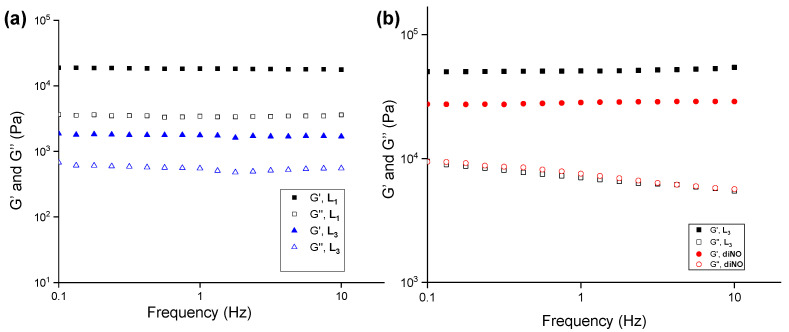
Frequency-sweep experiments performed on (**a**) **L_1_** and **L_3_** gels at 2.0 wt% and (**b**) **L_3_** and **diNO** gels at 4.0 wt% in water at a constant strain of 0.02% at 20.0 °C, respectively.

Frequency-sweep experiments were performed with **L_1_** and **L_3_** hydrogels (2.0 wt%) at a constant strain of 0.02% (within LVR) in a range of 0.1–10.0 Hz, which displayed constant elastic (G′) and viscous (G″) moduli under varying frequency. The hydrogel of **L_1_** displayed a higher elastic modulus (~10 times, Figure 1a) compared to the **L_3_** hydrogel. The enhanced mechanical stability can be correlated to the molecular structure of **L_1_**, which indicates that the 4-pyridyl functionality in **L_1_** plays a crucial role in the thermal and mechanical strength of the gel. The role of the relative position of the *N*-oxide moieties in the mechanical strength was analyzed by performing the frequency-sweep experiments on **L_3_** and isomeric **diNO** hydrogels at 4.0 wt%. The comparison of the elastic (G′) and viscous (G″) moduli of **L_3_** and isomeric **diNO** gels revealed that **L_3_** hydrogels displayed enhanced mechanical strength (~1.8-fold stronger) than the **diNO** hydrogels, which indicated that the mechanical strength of the gel network depended on the position of the pyridyl *N*-oxide moiety (Figure 1b).

We also performed the frequency-sweep experiments with **L_1_** and **L_3_** gels in DMSO/water and DMF/water (1:1, *v*/*v*) at 2.0 wt/v%, and the aqueous mixture gels of **L_1_** were stronger (2–3-fold) than the hydrogel of **L_1_** at 2.0 wt%. A similar trend was found for the aqueous mixture gels of **L_3_** in the DMSO/water gel (~2-fold stronger than **L_3_** hydrogels), but the DMF/water gel displayed similar mechanical strength as that of the hydrogel of **L_3_** at 2.0 wt% (Figure S2).

### 2.4. Scanning Electron Microscopy (SEM)

**Figure 2:**
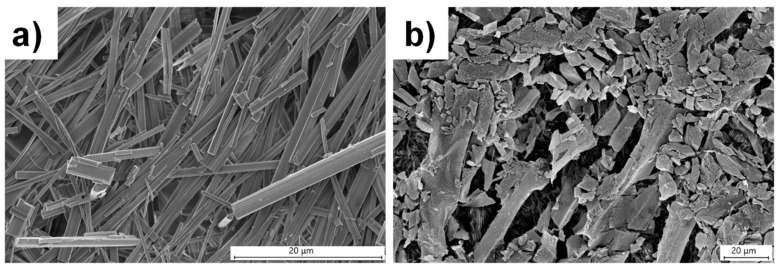
SEM images of xerogels in water with (**a**) **L_1_** and (**b**) **L_3_** at 2.0 wt%.

SEM is an important technique to visualize the morphology of gel fibers [12,67] and can be used to distinguish the self-assembly modes in supramolecular gels by analyzing the morphology of the gel fibers [68,69]. The morphologies of the fibrous network of **L_1_** and **L_3_** xerogels were analyzed by performing SEM on the dried gels in water at the minimum gelator concentration (1.8 wt%). **L_1_** displayed rod-like morphology with thickness ranging from 0.3–3.0 μm, but twisted rod-like morphologies were observed from **L_3_** xerogels, and the thickness of the fibers was within the range of 1.0–3.0 μm (Figure S3). SEM performed at 2.0 wt% (above MGC) on **L_1_** xerogels showed rod-like morphologies with thickness ranging from 0.5–4.0 μm (Figure 2a), but flake-like morphologies were observed for **L_3_**, with dimensions ranging from 4.0–16.0 μm (Figure 2b). The comparison of the SEM images of **L_3_** and isomeric **diNO** xerogels [55] from water at 4.0 wt% revealed that the morphologies of the gels did not depend on the relative position of the pyridyl *N*-oxide moiety (Figure S4).

We also analyzed the morphologies of **L_1_** and **L_3_** in the aqueous mixtures of DMSO and methanol (1:1, *v*/*v*) to evaluate the effect of solvents on the morphologies of gel fibers. The xerogel of **L_1_** prepared from DMSO/water and methanol/water (1:1, *v*/*v*, respectively) at 2.0 wt/v% displayed needle-shaped morphology with dimensions ranging from 0.5–6.0 μm (Figure S5). However, long rod-shaped morphology with fiber width ranging from 2.0 to 20.0 μm (Figure S6) was observed for **L_3_** xerogels in DMSO/water and methanol/water (1:1, *v*/*v*, respectively) at 2.0 wt/v%. These results indicate that morphologies of the fibrous network depend on the solvent system.

### 2.5. Structural Analysis

#### 2.5.1. Single Crystal X-ray Diffraction

**Figure 3:**
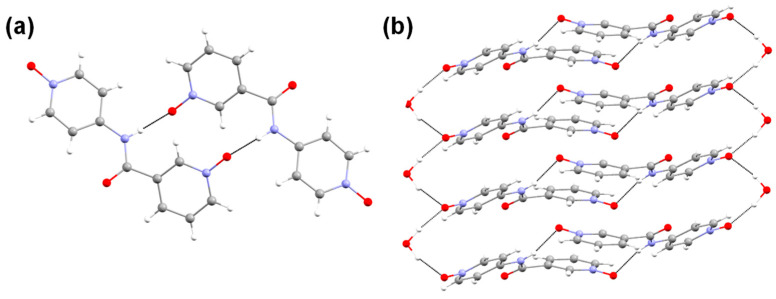
(**a**) Hydrogen-bonded dimer of **L_3_** stabilized by N–H···O interactions and (**b**) the aminopyridine *N*-oxide moiety interacting with the water molecule to form a 1D hydrogen-bonded chain of the dimers.

The slow evaporation of a dilute solution of **L_3_** resulted in X-ray-quality single crystals of **L_3_**. The analysis of the crystals using a polarized light microscope indicated that needle-shaped crystals with trace amounts of plate-shaped crystals were formed. The solid-state structural data were analyzed by single-crystal diffraction analysis, which helped us to correlate the key non-bonding interactions in the solid state and the gelation properties. The structural analysis of the needle-shaped crystals revealed that **L_3_** crystallized in a monoclinic space group (*P*2_1_/*c*) with a solvent water molecule (**L_3_**•H_2_O) (Figure 3a and Table S1). The N-H moiety of the amide group displayed hydrogen-bonding interaction with the *N*-oxide moiety of the nicotinamide moieties via N–H···O interactions (Table S2), resulting in a $R_{2}^{2}$ (14) hydrogen-bonded dimer [70] of **L_3_**. The oxygen atom of the aminopyridine *N*-oxide displayed a bifurcated hydrogen bonding with the solvent water molecule via O—H···O interactions, resulting in a 1D hydrogen-bonded chain of the dimers (Figure 3b). This 1D hydrogen-bonded chain can be considered as one of the crucial factors for supramolecular gelation [6,8] in LMWGs. The comparison of the crystal structure of **L_3_**•H_2_O with the isomeric **diNO** revealed the absence of such 1D hydrogen-bonded chains, which could be one of the factors in the better gelation ability and mechanical strength of **L_3_**•H_2_O compared to isomeric **diNO**.

We also analyzed the solid-state structure of the plate-shaped crystals (minor product), which indicated that **L_3_** crystallized in the monoclinic space group (*P*2/*c*) with two water molecules (**L_3_**•2H_2_O) (Figure S7 and Table S1). The molecule was planar compared to **L_3_**•H_2_O, but a similar interaction was observed between the amide and *N*-oxide moieties, resulting in a ($R_{2}^{2}$) (14) hydrogen-bonded dimer [70]. However, the aminopyridine *N*-oxide moiety displayed a bifurcated hydrogen bond with two water molecules, resulting in two-dimensional porous architecture (Figure S7b), which was further stabilized by various non-bonding interactions (Table S2).

#### 2.5.2. Powder X-ray Diffraction (PXRD)

**Figure 4:**
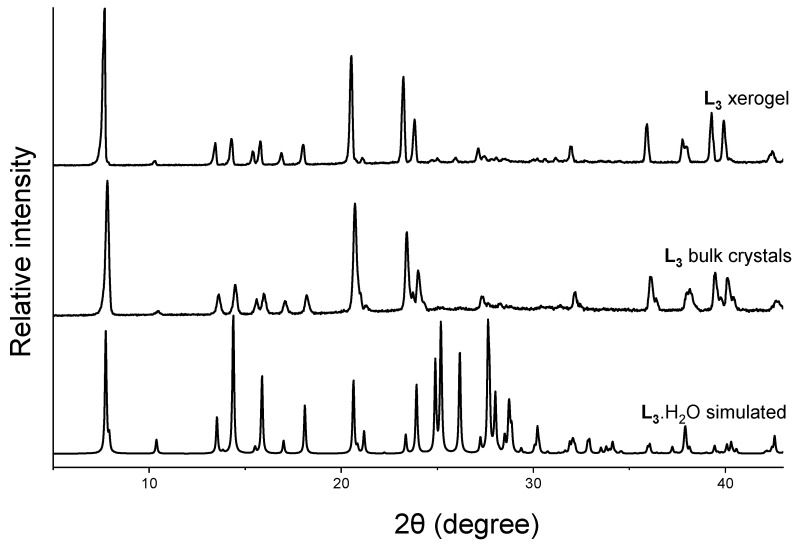
Comparison of the simulated pattern from single-crystal X-ray structure of **L_3_**•H_2_O with the PXRD pattern of the bulk crystals obtained from water and xerogel from water at 2.0 wt%.

Powder X-ray diffraction is a rapid and powerful tool to obtain an insight into the packing modes of solids. Comparing the PXRD pattern of the bulk material with the simulated pattern acquired from the single-crystal structure enabled us to evaluate the phase purity of the material [63,71]. We have shown that comparing the PXRD pattern of the xerogels with the simulated pattern of the gelator structure could provide information about the key interactions in the gel network architecture [6,12,32,55,72]. This method can be considered as an excellent strategy to correlate the self-assembly process in LMWGs despite the artefacts affecting the drying process [73]. We recorded the PXRD pattern of the **L_3_** crystals obtained via recrystallization from hot water (10.0 mg in 2.0 mL) and **L_3_** xerogel from pure water at 2.0 wt%. The PXRD pattern of the bulk crystals and the xerogel matched with the simulated pattern of the needle-shaped crystals **L_3_**•H_2_O (Figure 4), indicating that the hierarchical assembly of the xerogel network matched with the solid-state structure of **L_3_**•H_2_O. The simulated pattern of **L_3_**•2H_2_O did not match with the PXRD pattern of the bulk crystals and the xerogel, which indicated that the second form did not correspond to the structure of the xerogels (Figure S8).

### 2.6. Stimuli-Responsive Properties

LMWGs based on pyridyl-*N*-oxides display smart responses towards external stimuli because the molecular interactions of *N*-oxide moieties are based on partial charges, and the gelation can be turned ON/OFF in the presence of respective salts/ions. The cation/anions interact with gelator molecules constructively or destructively, depending on the electrostatic interaction and acidic/basic properties of the cations/anions [52,74]. The constructive interaction could trigger/enhance the gelation process [30,75,76], but a destructive interaction may lead to the dissolution/collapse of the gel network [27,39].

#### 2.6.1. Anion Sensing

We have previously reported the pyridyl-*N*-oxide-based compound’s sensing ability towards salts/ions [64,77] and have shown that cyanide ions can be detected using the gel-sol transition [62]. This prompted us to study the effect of salts/ions on the gelation properties of the *N*-oxide compounds of *N*-(4-pyridyl)nicotinamide. The stimuli-responsive properties of **L_1_** and **L_3_** in water were analyzed by treating the compounds at concentrations below MGC (at 1.5 wt%) with various halides of sodium and potassium ions (1.0 equiv.). Gel formation was observed for **L_1_** at 1.5 wt% in the presence of 1.0 equivalence of NaF, NaCl, NaBr, KF, KCl, KBr, and KI (Table S3). The experiments performed at a lower concentration of **L_1_** (1.2 wt%) resulted in selective gelation in the presence of 1.0 equivalence of KCl. The stimuli-responsive properties of **L_3_** in the presence of sodium and potassium halide salts (1.0 equiv.) displayed gel formation at 1.5 wt%. These results indicate the stimuli-responsive properties of **L_1_** and **L_3_** in water, where the salts/ions induce gel network formation (Table S3). The anion-sensing ability of **L_1_** and **L_3_** (1.5 wt%) was further analyzed in the presence of other anions, such as KNO_3_, KBF_4_, KPF_6_, and K_2_C_2_O_4_ (Table S3). Gels were obtained for all ions with **L_3_**, but a colloidal solution was observed with **L_1_** at 1.5 wt%. We compared the stimuli-responsive properties of **L_1_** and **L_3_** with isomeric *N*-oxides **INO** and **diNO**, respectively. The non-gelator **INO** (isomeric *N*-oxide of **L_1_**) did not form any gels in the presence of 1.0 equivalence of sodium and potassium salts in pure water at 1.5 wt%. The isomeric *N*-oxide of **L_3_ (diNO)** also failed to form gels at 1.5 wt%, which indicated that the effective interaction between the compound and the salts/ions was affected by the position of the *N*-oxide functionality. However, the **diNO** (MGC, 4.0 wt%) formed gels at a concentration below MGC (3.0 wt%) with these salts (1.0 equiv.).

**Figure 5:**
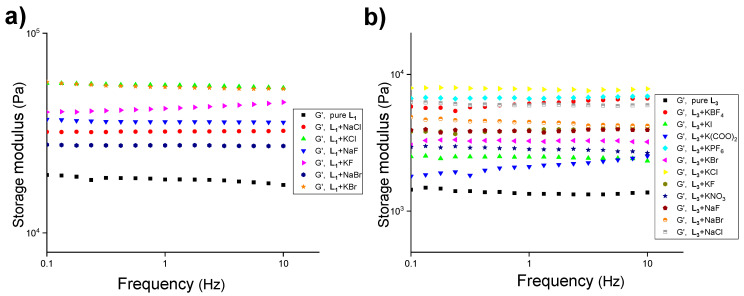
Frequency-sweep experiments of **L_1_** and **L_3_** at 1.5 wt% in water at 20.0 °C in the presence of various salts of sodium and potassium (1.0 equiv.) with a constant strain of 0.02%, (**a**) **L_1_** (pure ligand at 1.8 wt%), and (**b**) **L_3_** (pure ligand at 1.8 wt%).

The comparison of the mechanical strength of the gels (1.5 wt%) in the presence of halide salts of sodium and potassium with the hydrogels of **L_1_** and **L_3_** at MGC (1.8 wt%) revealed that enhanced mechanical strength was observed in both cases (Figure 5 and Table S4). A ~5.7-fold increase in the mechanical strength of **L_3_** (1.5 wt%) was observed in the presence of KCl and a ~4.3-fold increase with NaCl (Figure 5b). We further studied the effect of other anions (KNO_3_, KBF_4_, KPF_6_, and K_2_C_2_O_4_) with **L_1_** and **L_3_** (1.5 wt%) below MGC, and **L_1_** failed to form stable gels in the presence of these salts. However, the mixed gel of **L_3_** and the anions displayed enhanced mechanical strength compared to the **L_3_** hydrogels (Figure 5b and Table S4).

**Figure 6:**
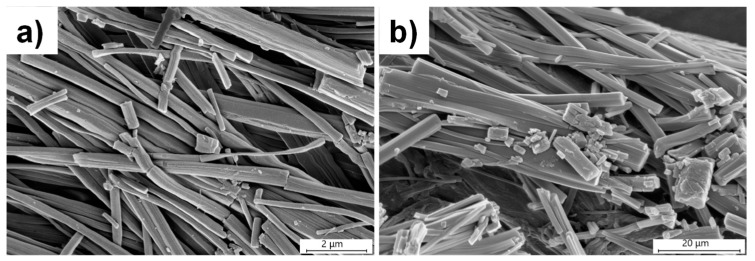
SEM images of xerogels obtained from pure water at 2.0 wt% in the presence of 1.0 equivalence of NaCl, (**a**) **L_1_**, and (**b**) **L_3_**.

The effect of anions on the morphology of the gel network was studied by analyzing the SEM images of the xerogels with anions. The xerogel of **L_1_** and **L_3_** in the presence of NaCl displayed fibrous morphologies with diameters ranging from 100 to 800 nm and 1.0 to 5.0 μm, respectively (Figure 6). The PXRD analysis of the xerogel of **L_3_** hydrogels at 1.5 wt% in the presence of 1.0 equivalence of NaCl and KCl showed a similar pattern with the pure xerogel of **L_3_** at 1.8 wt%, which suggested that the anions interacted with **L_3_** to form a stable gel below MGC without affecting the original network (Figure S9).

#### 2.6.2. Cation Sensing

**Figure 7:**
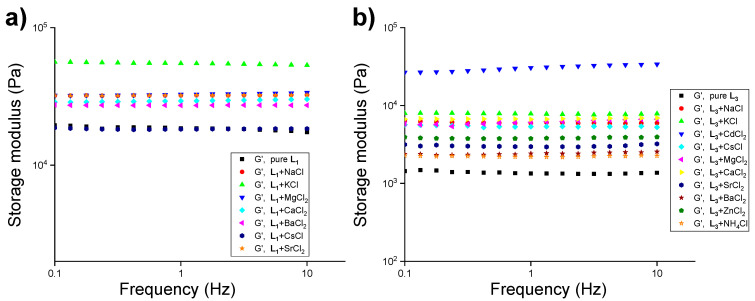
Frequency-sweep experiments with 1.5 wt% of the compound in the presence of various salts of chlorides (1.0 equiv.) in water at 20.0 °C with a constant strain of 0.02%, (**a**) **L_1_** (pure ligand at 1.8 wt%), and (**b**) **L_3_** (pure ligand at 1.8 wt%).

The gelation property was observed to be independent of the size and nature of the anions, which prompted us to study the stimuli-responsive properties of **L_1_** and **L_3_** with chloride salts having various countercations (1.0 equiv.) at a concentration below MGC (1.5 wt%). **L_1_** formed a stable gel in the presence of 1.0 equivalence CsCl, MgCl_2_, CaCl_2,_ SrCl_2_, and BaCl_2_, but no gels were observed in the presence of 1.0 equivalence of NH_4_Cl. On the other hand, **L_3_** formed a gel in the presence of all of the above-mentioned chloride salts (Table S5). The mechanical strength of **L_1_** and **L_3_** with these salts was evaluated to observe the effect of cations in gel network formation. Analysis of the results indicated that the addition of chloride salts of magnesium, calcium, strontium, and barium enhanced the mechanical strength (1.5–3.0-fold) of **L_1_** compared to **L_1_** (1.8 wt%) hydrogel (Figure 7a and Table S6). A similar trend was observed for **L_3_** gels in the presence of the chloride salts of magnesium, calcium, strontium, barium, cesium, and ammonium (Figure 7b and Table S6).

The cation-sensing ability of the isomeric *N*-oxide compounds (**INO** and **diNO**) were compared with **L_1_** and **L_3_** gels in the presence of various cations. Gelation was not observed for both **INO** and **diNO** at 1.5 wt% in the presence of MgCl_2_ and CaCl_2_ (1.0 equiv.) However, **diNO** formed stable hydrogel at 3.0 wt% (below MGC) with 1.0 equivalence MgCl_2_ and CaCl_2_. The comparison of the sensing ability of the isomeric *N*-oxides confirms that the relative position of *N*-oxide plays a crucial role in cation sensing. SEM performed on the dried gels of **L_1_** and **L_3_** at 2.0 wt% in the presence of MgCl_2_ (1.0 equiv.) revealed that the morphology of the xerogels was identical to the xerogels of **L_1_** and **L_3_** hydrogels (2.0 wt%, Figure S10). PXRD studies with the chloride salts of magnesium and calcium (1.0 equiv) at 1.5 wt% of **L_3_** showed no change compared to the powder pattern of the xerogel of **L_3_** at 1.8 wt%, suggesting similar molecular packing (Figure S11). The molecular interactions of *N*-oxide moieties are based on partial charges, and the enhanced gelation property in the presence of anions/cations may be attributed to the favorable interactions between the *N*-oxide moieties and anions/cations, which is presumably due to the combination of the non-bonding and ionic interactions. The enhanced mechanical properties of **L_1_** and **L_3_** in the presence of various cations prompted us to study the stimuli-responsive properties of **L_1_** and **L_3_** with transition metal salts, which could form metallogels.

Metallogels based on transition metals are multi-responsive soft materials with intriguing potential applications in cosmetics, food processing, drug delivery, and catalysis [78,79]. Metal-based supramolecular gels are formed by the addition of metal ions to a gelator/non-gelator, and the interaction of the metal salt with the gelator could induce/enhance gelation properties to form metallogels [52,79]. However, the addition of metal salts could disrupt the key interactions in the gel network, leading to the dissolution of the gel network [52,79]. The metal-coordination-driven self-assembly plays a key role in the formation of a gel network, which is supported by various non-covalent interactions [80,81]. The presence of the coordinating functionality (pyridyl *N*-oxide moiety) and a hydrogen-bonding group (amide moiety) in **L_1_** and **L_3_** will make these gelators ideal candidates for metal-based supramolecular gels [52].

**Figure 8:**
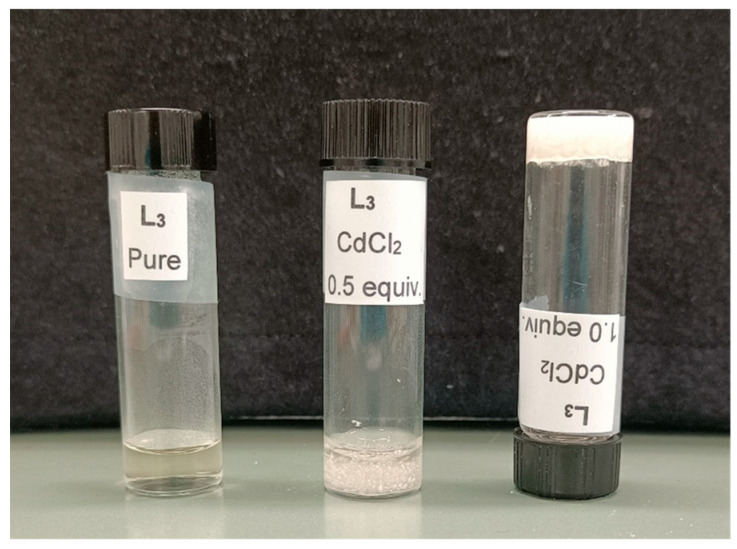
Stimuli-responsive behavior of **L_3_** at 0.7 wt% in the presence of CdCl_2_.

The effect of transition metal salts on the stimuli-responsive property of the hydrogel was studied by adding one equivalent of the metal salt (CuCl_2_, ZnCl_2_, or CdCl_2_) to **L_1_** or **L_3_** at 1.5 wt%. Gels were not obtained for **L_1_** in the presence of these transition metal chlorides. However, **L_3_** gelled at 1.5 wt% in the presence of ZnCl_2_ and CdCl_2_ (1.0 equiv.), but no gel was observed with CuCl_2_. We repeated the experiments by lowering the concentration of **L_3_** (0.7 wt%), and selective gel formation was observed with a 1.0 equivalence of CdCl_2_, highlighting the specific sensing of cadmium chloride at this concentration (Figure 8). The **L_3_**-CdCl_2_ gelator obtained at 0.7 wt% of **L_3_** and CdCl_2_ (1.0 equiv.) was stable for several weeks, and the experiments performed at lower concentrations of CdCl_2_ (0.5 equiv.) resulted in precipitation (Figure 8). We further analyzed the gelation behavior of **L_3_** in the aqueous mixtures (1:1, *v*/*v*) of methanol, ethanol, DMF, and DMSO at 2.0 wt/v% in the presence of 1.0 equivalence of ZnCl_2_ and CdCl_2_. We observed gelation with ZnCl_2_ and CdCl_2_ in the aqueous mixtures of DMF/DMSO, but gels were formed with CdCl_2_ in MeOH/water and EtOH/water. The gelation ability of **INO** with ZnCl_2_ and CdCl_2_ was previously reported by our group [62]. The isomeric **diNO** formed a precipitate below 2.5 wt% with 1.0 equivalence of CuCl_2_ or CdCl_2_, which highlighted the effective gelation ability of **L_3_** over the isomeric gelator **diNO** in the presence of transition metals. However, hydrogels were formed at 2.5 wt% (below MGC) with 1.0 equivalence of CuCl_2_ or CdCl_2_, and a precipitate was formed in the presence of ZnCl_2_.

**Figure 9:**
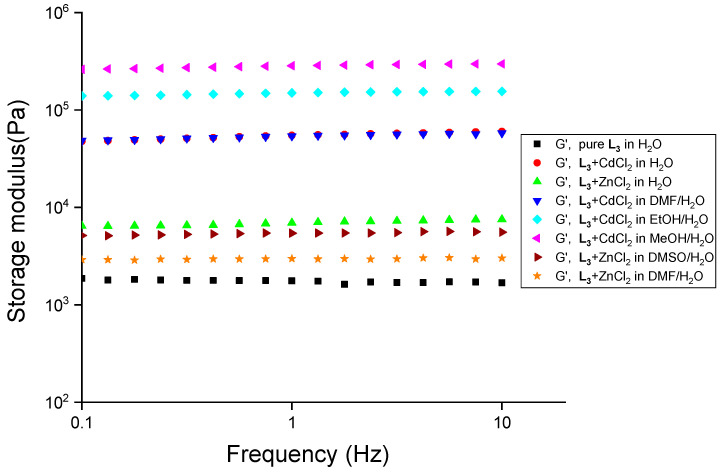
Frequency-sweep experiments with **L_3_** at 2.0 wt% and in the presence of chloride salts of zinc and cadmium (1.0 equiv.) in various solvent mixtures at 20.0 °C with a constant strain of 0.02%.

The mechanical strength of the gels with transition metals was analyzed by performing frequency-sweep experiments, and an abrupt increase in the mechanical strength (~24.7 fold) was observed for **L_3_** (1.5 wt%) with CdCl_2_ (1.0 equiv.). We studied the mechanical strength of the gels at 2.0 wt/v% of **L_3_** with CdCl_2_ in water and a 1:1 (*v*/*v*) aqueous mixture of methanol, ethanol, DMSO, and DMF. A steep increase in the mechanical strength was observed for **L_3_**-CdCl_2_ gels in water (~35.6-fold), methanol/water (~175-fold), ethanol/water (~91.7-fold), and DMF/water (~34.3-fold) with **L_3_** (2.0 wt/v%) (Figure 9).

Similarly, we observed an enhanced mechanical strength for **L_3_** gels at 2.0 wt% with 1.0 equivalence of ZnCl_2_ in water (~4.4-fold), DMF/water (~1.8-fold), and DMSO/water (~3.3-fold) (Figure 9).

**Figure 10:**
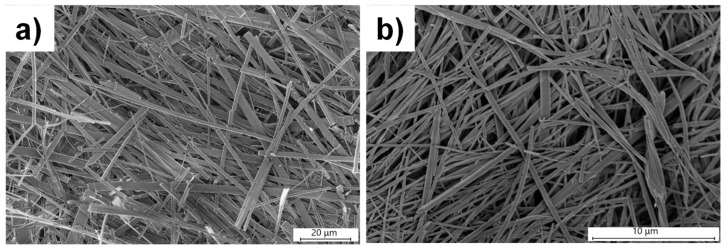
SEM images of xerogels obtained with **L_3_** in the presence of 1.0 equivalence of CdCl_2_ in (**a**) 0.7 wt% in pure water and (**b**) 2.0 wt/v% in DMSO/water (1:1, *v*/*v*).

The morphological analysis of the dried gels at 2.0 wt% of **L_3_** with 1.0 equivalence of CdCl_2_ from water revealed rod-shaped morphology with fiber width ranging from 0.4 to 4.0 μm (Figure S12). Long needle-shaped fibers were observed for dried gels at 0.7 wt% of **L_3_** with 1.0 equivalence of CdCl_2_, and the diameter of the fibers ranged from 1.0 to 6.0 μm (Figure 10a). The xerogels obtained from DMSO/water (1:1, *v*/*v*) at 2.0 wt% of **L_3_** and CdCl_2_ (1.0 equiv.) displayed similar fibrous morphology, with a fiber width range of 0.1–1.0 μm (Figure 10b).

We analyzed the powder X-ray patterns of the dried gels at 2.0 wt% and 0.7 wt% of **L_3_** hydrogels in the presence of 1.0 equivalence of CdCl_2_, respectively. Analysis of the PXRD pattern revealed a different PXRD pattern for mixed **L_3_**-CdCl_2_ xerogels compared to the **L_3_** xerogel at 2.0 wt% (Figure S13) in both cases. This may be attributed to the complexation of **L_3_** with CdCl_2_, resulting in the formation of a stable hydrogel at a lower ligand concentration (0.7 wt%) induced by metal-ligand-driven supramolecular self-assembly. PXRD pattern of the mixed **L_3_**-CdCl_2_ xerogels at 2.0 wt/v% of **L_3_** obtained from an aqueous mixture (1:1, *v*/*v*) of methanol, ethanol, DMF, and DMSO did not match with the xerogel of **L_3_**, which confirmed that interaction of **L_3_** with CdCl_2_ was independent of solvent composition. However, the PXRD patterns of mixed **L_3_**-CdCl_2_ in different solvent mixtures were not similar, because the crystal packing was affected by the nature of the solvent (Figure S13). The morphology, PXRD pattern, and gel-state properties of **L_3_**-CdCl_2_ gels suggest that **L_3_** interacts with CdCl_2_, which may be considered as metallogels. PXRD studies with ZnCl_2_ (1.0 equiv) at 1.5 wt% of **L_3_** in water and in aqueous mixtures (1:1, *v*/*v*) of DMF and DMSO matched with the xerogel of **L_3_** at 1.8 wt%, indicating similar solid-state structure (Figure S14). This suggests that ZnCl_2_ exists as non-coordinated metal salts in the gel network, and the gelation properties can be explained based on the fact that metal nanoparticles and non-coordinated metal complexes are known to enhance the gelation properties of LMWGs [82,83,84,85].

## 3. Conclusions

The role of the relative position of functional groups in the gelation properties of LMWGs was studied by analyzing the gelation properties of isomeric mono-/bis-pyridyl-*N*-oxide compounds. We synthesized mono-/bis-pyridyl-*N*-oxide compounds of *N*-(4-pyridyl)nicotinamide (**L_1_**-**L_3_**), and the gelation properties of **L_1_** and **L_3_** were compared with isomeric *N*-oxide compounds (**INO** and **diNO**, respectively). Gelation tests revealed that **L_1_** and **L_3_** formed hydrogels, whereas **L_2_** was a non-gelator, which underlines the importance of the nicotinamide *N*-oxide moiety in gel network formation. Comparing the gelation behavior of **L_1_** and **L_3_** with corresponding isomeric *N*-oxide compounds (**INO** and **diNO**) revealed that the relative position of the *N*-oxide moieties played a crucial role in the self-assembly process of LMWGs. SEM analysis revealed that the morphology was independent of the relative position of the pyridyl *N*-oxide moiety. Single-crystal X-ray diffraction of **L_3_** revealed the existence of a 1D hydrogen-bonded chain, which was crucial for gel network formation. The solid-state structure was correlated to the xerogels to obtain an insight into the key interactions responsible for gel network formation. The stimuli-responsive properties of **L_1_**–**L_3_** were studied with various salts/ions, and anion-induced gelation was observed for **L_1_** and **L_3_** in the water below MGC with **L_1_** and **L_3_** in water. **L_3_** was very versatile in nature, as it formed a gel below MGC with most of the salts or transition metal salts, which showed the cooperative interaction between the *N*-oxide and salts/ions. An abrupt increase in the mechanical property of **L_3_** was observed in the presence of 1.0 equivalence of cadmium chloride, and a stable gel was formed at a very low concentration of the gelator (0.7 wt%). The effective sensing of cadmium chloride may be due to the metal-ligand coordination-driven supramolecular assembly. We showed that the salt-induced gelation depended on the nature and the position of the functional group, which will open the door for designing LMWGs based on *N*-oxide moieties with intriguing features.

## 4. Materials and Methods

The starting materials and solvents were obtained from Sigma-Aldrich (MEDOR ehf, Reykjavik, Iceland) and TCI-Europe (Boereveldseweg, Belgium) and utilized as provided. Deionized water was used to perform the gelation studies. The NMR spectra (^1^H and ^13^C, Figures S15–S20) were recorded with a Bruker Avance 400 spectrometer (Rheinstetten, Germany), and the scanning electron microscopy (SEM) images were captured with a Leo Supra 25 microscope (Carl Zeiss, Oberkochen, Germany). The mechanical strength evaluation was performed in an Anton Paar’s MCR 302 (Graz, Austria) modular compact rheometer. Single-crystal X-ray diffraction (SCXRD) and powder X-ray diffraction (PXRD) experiments were carried out using a Bruker D8 venture (Karlsruhe, Germany) and PANalytical instrument (Almelo, Netherlands), respectively. We synthesized the *N*-oxide compounds by replacing the pyridyl group of *N*-(pyridin-4-yl)nicotinamide (**4PNA**) with pyridyl *N*-oxides [45]. Synthesis of 3-carboxypyridine 1-oxide [86] and 4-aminopyridine 1-oxide [87] was performed following literature and confirmed by matching the analytical data with the reported compounds. The INO and **diNO** compounds were synthesized by following the reported procedure from our previous work [55].

### 4.1. Synthesis of Ligands

#### 4.1.1. Synthesis of 3-(pyridin-4-ylcarbamoyl) pyridine 1-oxide (L_1_)

To a 100.0 mL two-neck round bottom flask we added 3-carboxypyridine 1-oxide (4.0 g, 30.0 mmol) with 20.0 mL of thionyl chloride, and the solution was refluxed overnight. The solvents were evaporated to dryness, and the corresponding acid chloride formed was used for the next step without further purification. Anhydrous DMF (around 25.0 mL) was added into the flask, followed by 4-aminopyridine (2.7 g, 28.7 mmol), and the mixture was cooled in an ice bath to 0 °C with constant stirring. A solution of triethylamine (4.5 mL) in 10.0 mL DMF was added dropwise to the reaction mixture at 0 °C over an hour, and the resulting yellow colloidal solution was stirred at room temperature overnight. To this mixture, water was added, resulting in a thick precipitate, which was filtered. The precipitate was then stirred in a 5.0% NaHCO_3_ solution for 4.0 h and was filtered. The residue was washed with an excess of cold water, air-dried, and recrystallized from hot water to obtain the product. Yield 70.0%. ^1^H NMR (400 MHz, DMSO-*d_6_*) δ (ppm): δ 10.80 (s, 1H), 8.74 (s, 1H), 8.51 (d, J = 5.8 Hz, 2H), 8.43 (d, J = 6.6 Hz, 1H), 7.81 (d, J = 8.0 Hz, 1H), 7.74 (d, J = 6.0 Hz, 2H), 7.59 (t, J = 7.3 Hz, 1H). ^13^C {^1^H} NMR (101 MHz, DMSO-*d_6_*) δ 162.65, 150.46, 145.27, 141.29, 138.02, 133.63, 126.58, 124.49, 114.11. HRMS (APCI): C_11_H_10_N_3_O_2_ [M + H]^+^, 216.0768; found, 216.0765.

#### 4.1.2. Synthesis of 4-(nicotinamido) pyridine 1-oxide (L_2_)

Compound **L_2_** was synthesized by following a similar procedure used for compound **L_1_**. Nicotonic acid (1.0 g, 8.12 mmol), thionyl chloride (20.0 mL), 4-aminopyridine 1-oxide (0.89 g, 8.12 mmol), and triethylamine (2.26 mL) in 30.0 mL DMF. Yield 68.0%. ^1^H NMR (400 MHz, DMSO-*d_6_*) δ 10.96 (s, 1H), 9.11 (s, 1H), 8.78 (dd, *J* = 4.8, 1.6 Hz, 1H), 8.32–8.28 (m, 1H), 8.19 (d, *J* = 7.5 Hz, 2H), 7.83 (d, *J* = 7.5 Hz, 2H), 7.60–7.56 (m, 1H). ^13^C {^1^H} NMR (101 MHz, DMSO-*d_6_*) δ 164.48, 152.59, 148.79, 138.92, 136.31, 135.64, 129.84, 123.61, 116.86. HRMS (APCI): C_11_H_9_N_3_NaO_2_ [M + Na]^+^, 238.0587; found, 238.0594.

#### 4.1.3. Synthesis of 3-((1-oxidopyridin-4-yl) carbamoyl) pyridine 1-oxide (L_3_)

To a 100.0 mL round-bottomed flask, *N*-(pyridin-4-yl) nicotinamide (2.0 g, 10.0 mmol) and MeOH (40.0 mL) were added to dissolve and stirred. 3-Chloroperoxybenzic acid (6.8 g, 40.0 mmol) was added in portions over 15.0 min to the solution and was refluxed overnight. The solution was filtered, and the precipitate was washed with 5.0% sodium bicarbonate and thrice with cold water. The precipitate was further recrystallized from hot water. Yield 55.0%. ^1^H NMR (400 MHz, DMSO-*d_6_*) δ 10.90 (s, 1H), 8.73 (s, 1H), 8.42 (m, 1H), 8.19 (d, *J* = 7.6 Hz, 2H), 7.81 (m, 1H), 7.79 (d, *J* = 7.6 Hz, 2H), 7.61–7.57 (m, 1H). ^13^C {^1^H} NMR (101 MHz, DMSO-*d_6_*) δ 162.01, 141.31, 138.94, 137.92, 135.69, 133.50, 126.63, 124.45, 116.97. HRMS (APCI): C_11_H_9_N_3_O_2_ [M + Na]^+^, 254.0536; found, 254.0540.

### 4.2. Gelation Studies

Hydrogelation ability was evaluated with all of the compounds by weighing different amounts (ranging from 10.0 to 40.0 mg) of the compound in a standard 7.0 mL vial; 1.0 mL of water was added, and the vial was sealed. The vial containing the mixture was then sonicated and slowly heated to obtain a clear solution, which was then left undisturbed for 24.0 h. Gelation was confirmed via a vial inversion test. Gelation tests were also performed in aqueous mixtures by dissolving the compound in 0.5 mL of distilled water and 0.5 mL of an appropriate solvent, and the vial was sealed. The mixture was sonicated, heated to obtain a clear solution, and checked for gelation ability. Gelation tests were performed in the presence of various salts with 15.0 mg of the compounds (**L_1_** and **L_3_**) in 1.0 mL of water followed by the addition of 1:1 molar equivalent of an appropriate salt. The mixture was sonicated and heated after sealing the vial to obtain a transparent solution. The solution was left undisturbed, and a vial inversion test confirmed gel formation. The experiments were repeated three times to confirm the results.

#### 4.2.1. Minimum Gelator Concentration (MGC)

MGC was performed in deionized water/aqueous mixtures by weighing various concentrations of the compounds in a standard 7.0 mL vial and adding 1.0 mL of deionized water/aqueous mixtures. The corresponding mixture was sonicated and gradually heated to dissolve the compounds, and the solution was kept at room temperature for gel formation. The minimum amount of the compound required to form a stable gel after 24.0 h was recorded as the MGC.

#### 4.2.2. *T_gel_* Experiments

The necessary amount of gelator and 1.0 mL of solvent were added to a 7.0 mL standard vial. After sonication, the mixture was heated to obtain a clear solution, and the mixture was left undisturbed to gel. A ball-drop method was used to observe the gel-to-solution transition temperature after 24.0 h (*T_gel_*). A spherical glass ball was carefully positioned on top of the gel and the vial was immersed in an oil bath; a thermometer and a magnetic stirrer were equipped to check the temperature. The oil bath was gradually heated at 10.0 °C per minute. The glass ball slowly became immersed into the gel as the temperature increased, and the temperature at which the ball touched the bottom of the vial was recorded as *T_gel_*.

### 4.3. Rheology

A 2.5 cm stainless steel parallel plate geometry configuration was used to perform the rheological measurements. In all cases, oscillatory measurements were conducted at a constant temperature of 20.0 °C. To maintain a constant temperature of 20.0 °C and to prevent solvent loss for amplitude and frequency sweeps, a Peltier temperature control hood was employed. Gels were prepared by dissolving an appropriate amount of gelator in 1.0 mL of solvent/solvent mixtures. After 24 h, amplitude-sweep experiments were carried out by adding approximately ~1.0 mL of gel to the plate. The frequency was maintained at 1.0 Hz during the amplitude sweep with log ramp strain (Ƴ) ranges of 0.01–100%. The frequency-sweep experiments were carried out between 0.1 and 10.0 Hz within the linear viscoelasticity domain (0.02% strain). The experiments were also performed in the presence of various salts at 1:1 molar equivalent in water with a similar procedure as mentioned above.

### 4.4. Scanning Electron Microscopy (SEM)

The surface morphologies of the xerogels were analyzed on a Leo Supra 25 microscope. Gels of **L_1_** and **L_3_** were prepared in water at 2.0 wt%. We also prepared the aqueous mixture gels of **L_3_** at 2.0 wt%. The gels were filtered after 24.0 h and dried under a fume hood to obtain the xerogel. A small part of xerogel was placed on a pin mount with the carbon tab on top, coated with gold for 5.0–6.0 min (~15.0 nm thickness) to avoid surface charging, and pictures were acquired at 3.0 kV with a working distance of 3–4 mm. An in-lens detector captured the SEM images. SEM of the xerogel of gelator **L_1_** in the presence of sodium, magnesium, and calcium salts and **L_3_** in the presence of sodium, magnesium, zinc (II), and cadmium (II) salts were also recorded.

### 4.5. Single-Crystal X-ray Diffraction

Crystals of compound **L_3_** were obtained by the slow evaporation of 10.0 mg of the compound in 3.0 mL of water to obtain needle- and block-shaped crystals. X-ray analysis was performed on a Bruker D8 Venture (Photon100 CMOS detector) diffractometer provided with Cryostream (Oxford Cryosystems) open-flow nitrogen cryostats. The data were collected using MoKα radiation (λ = 0.71073 Å) for the plate-shaped crystals at 296(2) K and CuK_α_ radiation (λ = 1.542 Å) for the needle-shaped crystal at 302(2) K. Apex III software (Bruker AXS: Madison, WI, USA, 2015) was utilized for the unit cell determination, data collection, data reduction, structure solution/refinement, and empirical absorption correction (SADABS). A direct method was used for solving the structure and was refined by the full-matrix least-squares on F^2^ for all data using SHELXTL version 2017/1 [88]. All non-disordered non-hydrogen atoms were refined anisotropically, and all of the hydrogen atoms were placed in the calculated positions and refined using a riding model, except for solvent water molecules. The hydrogen atoms of water molecules were located on the Fourier map and refined. The crystallographic data and hydrogen-bonding parameters are given in Tables S1 and S2 (see Supplementary Materials). The crystallographic data were deposited at the Cambridge Crystallographic Data Centre and can be obtained free of charge, and the CCDC numbers are 2226289–2226290.

### 4.6. Powder X-ray Diffraction (PXRD)

The bulk crystals of compound **L_3_** were obtained by the slow evaporation of the solution of **L_3_** (20.0 mg in 3.0 mL water). The crystals were filtered, dried in the air, and ground to a fine powder. The xerogels of **L_3_** were prepared from water and the aqueous mixtures, following a similar procedure as mentioned above. We also performed PXRD of the dried gels at 2.0 wt/v% in various solvents/solvent mixtures obtained by adding different salts at 1:1 molar equivalent. All experiments were performed on a PANalytical device with a Cu anode, 2θ from 5.0 to 60.0°, and a 0.02 step size.

## Figures and Tables

**Table 1:** Minimum gelator concentration and ***T_gel_*** values of **L_1_** and **L_3_** (2.0 wt/v%) in water and aqueous mixtures.

	L_1_		L_3_	
Solvents	MGC (wt/v%)	*T_gel_* (°C)	MGC (wt/v%)	*T_gel_* (°C)
Water	1.8	128.0	1.8	76.9
MeOH/water	2.0	77.1	2.0	69.7
EtOH/water	2.0	73.2	2.0	63.3
DMF/water	2.0	76.5	2.0	74.3
DMSO/water	2.0	87.9	2.0	94.2

## Data Availability

Not applicable.

## Supplementary Materials

The following supporting information can be downloaded at: https://www.mdpi.com/article/10.3390/gels9020089/s1, Scheme S1. Synthesis of **L_1_**, Scheme S2. Synthesis of **L_2_**, Scheme S3. Synthesis of **L_3_**. Figure S1. Amplitude-sweep experiments with gels of **L_1_** and **L_2_** (2.0 wt%) in water at 20.0 °C with a constant frequency of 1.0 Hz, Figure S2. Frequency-sweep experiments with gels of **L_1_** and **L_3_** (2.0 wt%) in aqueous mixtures at 20.0 °C with a constant strain of 0.02%, Figure S3. SEM images of (a) **L_1_** and (b) **L_3_** xerogels in water at 1.8 wt%, Figure S4. SEM images of the xerogels of (a) **L_3_** and (b) **diNO** gels obtained from water at 4.0 wt%, Figure S5. SEM images of **L_1_** xerogels in (a) DMSO/water (1:1, *v*/*v*) and (b) methanol/water (1:1, *v*/*v*) at 2.0 wt/v%, Figure S6. SEM images of **L_3_** xerogels from (a) DMSO/water (1:1, *v*/*v*), and (b) methanol/water (1:1, *v*/*v*) at 2.0 wt/v%, Figure S7. (a) Molecular structure of **L_3_**•2H_2_O and (b) two-dimensional hydrogen-bonded network with water molecules (space fill model) located in the cavity, Figure S8. Comparison of the simulated pattern of the single-crystal X-ray structure of **L_3_**•2H_2_O with the PXRD pattern of the bulk crystals obtained from water, and xerogel from water at 2.0 wt%, Figure S9. PXRD pattern of xerogels obtained from the hydrogel of **L_3_** at 2.0 wt% and in the presence of 1.0 equivalence of NaCl and KCl, Figure S10. SEM images of xerogels of (a) **L_1_** and (b) **L_3_** at 2.0 wt% obtained from water in the presence of 1.0 equivalence of MgCl_2_, Figure S11. Comparison of the PXRD pattern of xerogels (2.0 wt%) of **L_3_** hydrogels and the gels in the presence of 1.0 equivalence of MgCl_2_ and CaCl_2_, Figure S12. SEM images of the xerogels of **L_3_** in the presence of 1.0 equivalence of CdCl_2_ in water at 2.0 wt%, Figure S13. Comparison of the PXRD pattern of **L_3_** xerogel with the PXRD pattern of the xerogels of **L_3_**-CdCl_2_ mixture in various solvents, Figure S14. Comparison of the PXRD pattern of **L_3_** xerogel from water with the PXRD pattern of the xerogels of the mixture (**L_3_** + ZnCl_2_) in various solvents, Figure S15. ^1^H NMR spectrum of compound **L_1_**, Figure S16. ^13^C NMR spectrum of compound **L_1_**, Figure S17. ^1^H NMR spectrum of compound **L_2_**, Figure S18. ^13^C NMR spectrum of compound **L_2_**, Figure S19. ^1^H NMR spectrum of compound **L_3_**, Figure S20. ^13^C NMR spectrum of compound **L_3_**. Table S1: Crystal data, Table S2: Hydrogen-bonding parameters, Table S3: Stimuli-responsive properties of the gelators **L_1_** and **L_3_**: Anion sensing in water at 1.5 wt%, Table S4: Increase in G’ values of the gelators at 1.5 wt% in the presence of various sodium and potassium salts in comparison with the hydrogels (1.8 wt%), Table S5: Stimuli-responsive properties of the gelators **L_1_** and **L_3_**: Cation sensing in water at 1.5 wt%, Table S6: Increase in G’ values of the gelators at 1.5 wt% in the presence of chloride salts of various cations in comparison with the hydrogels (1.8 wt%).
