# Supplementary material for: Stimuli-Responsive Properties of Supramolecular Gels Based on Pyridyl-N-oxide Amides
Source: Gels. 2023 Jan 20;9(2):89. doi: 10.3390/gels9020089 (PMC9956205; doi:10.3390/gels9020089)
Supplement: Supplementary file 1 [file gels-09-00089-s001.zip › gels-2153709-supplementary.pdf]

Supporting Information

Stimuli-responsive Properties of Supramolecular Gels based on Pyridyl-*N*-oxide Amides

Sreejith Sudhakaran Jayabhavan [1], Baldur Kristinsson [1], Dipankar Ghosh [1], Charlène Breton [1], and Krishna K. Damodaran [1,*]

Table of contents

**1.**	Synthetic scheme	2
**2.**	Rheology	3
**3.**	Scanning electron microscopy	4
**4.**	X-ray crystallography	8
**5.**	Physical properties in the presence of salts....................................	11
**6.**	NMR spectra	16

1. Synthetic scheme

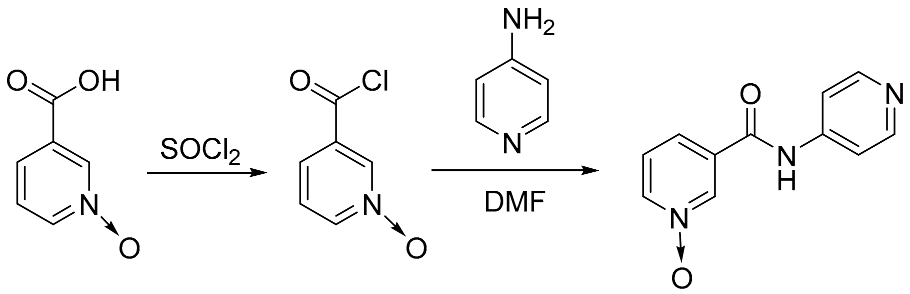

Scheme S1. Synthesis of $\mathbf{L_1}$

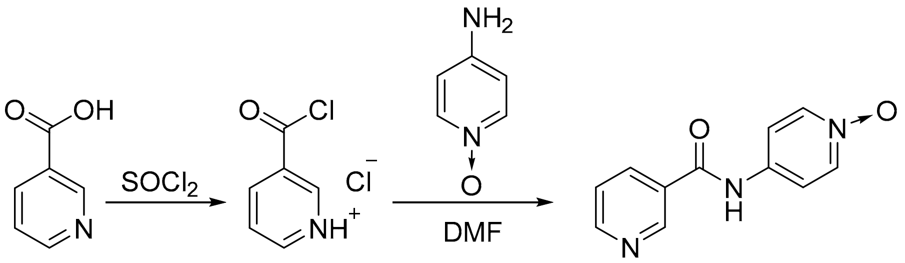

Scheme S2. Synthesis of $\mathbf{L_2}$

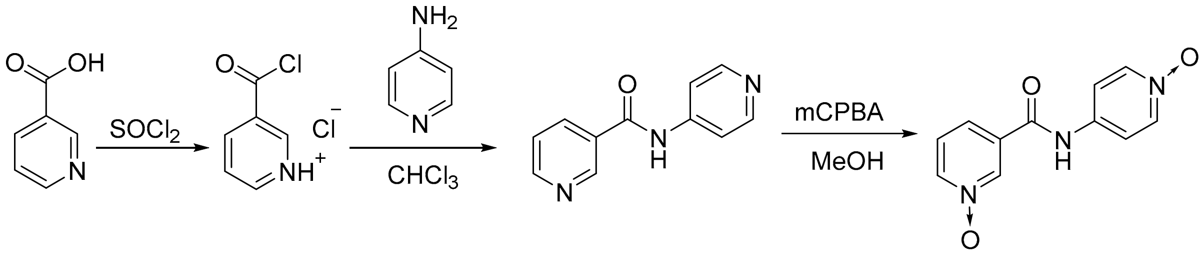

Scheme S3. Synthesis of $\mathbf{L_3}$

2. Rheology

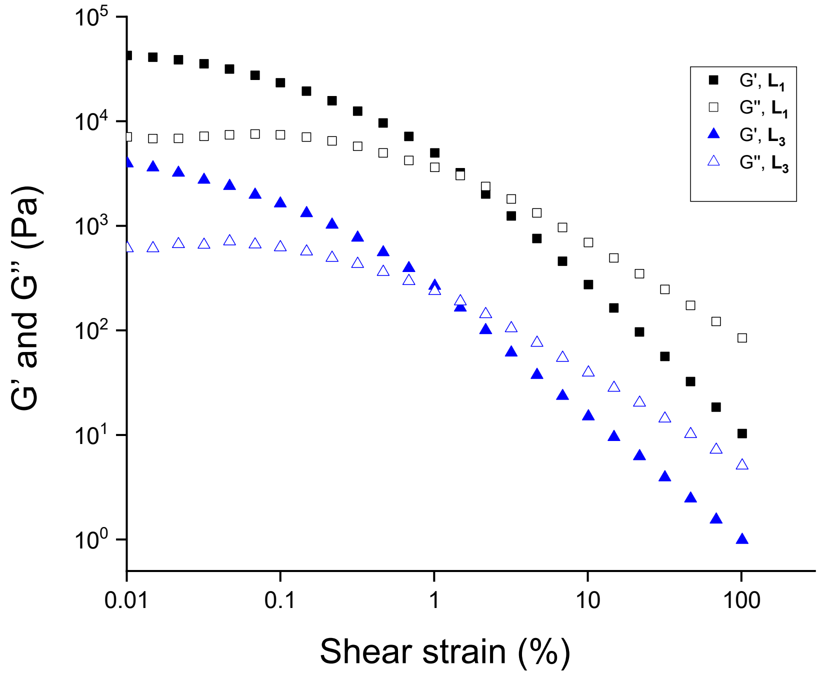

Figure S1. Amplitude-sweep experiments with gels of $\mathbf{L_1}$ and $\mathbf{L_2}$ (2.0 wt%) in water at 20.0 °C with a constant frequency of 1.0 Hz.

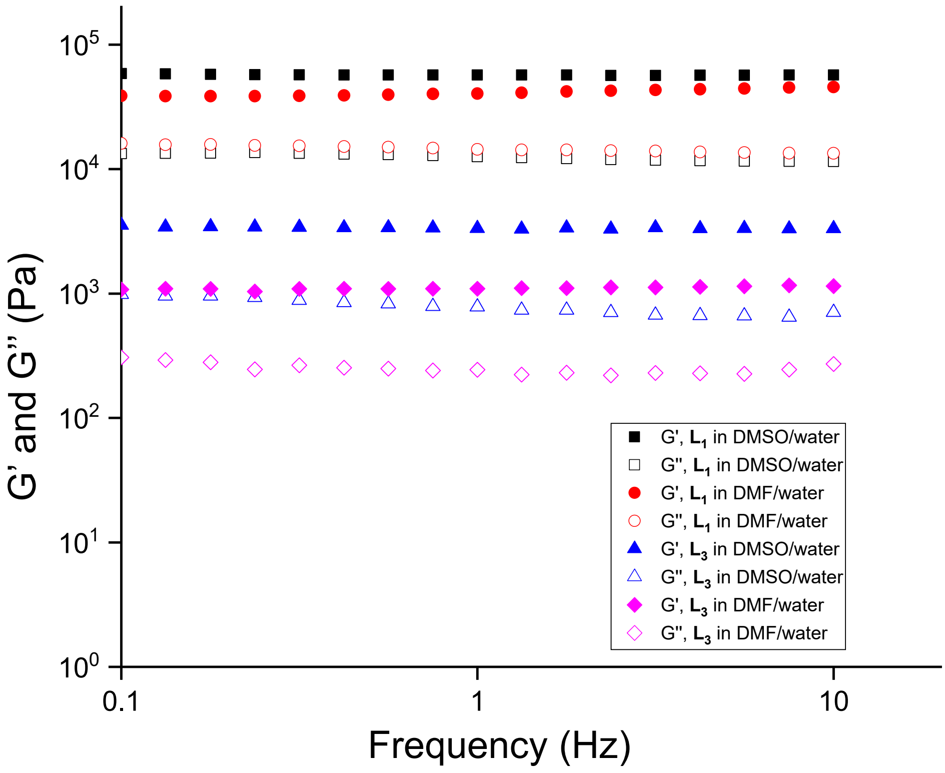

Figure S2. Frequency-sweep experiments with gels of $\mathbf{L_1}$ and $\mathbf{L_3}$ (2.0 wt%) in aqueous mixtures at 20.0 °C with a constant strain of 0.02%.

3. Scanning electron microscopy (SEM)

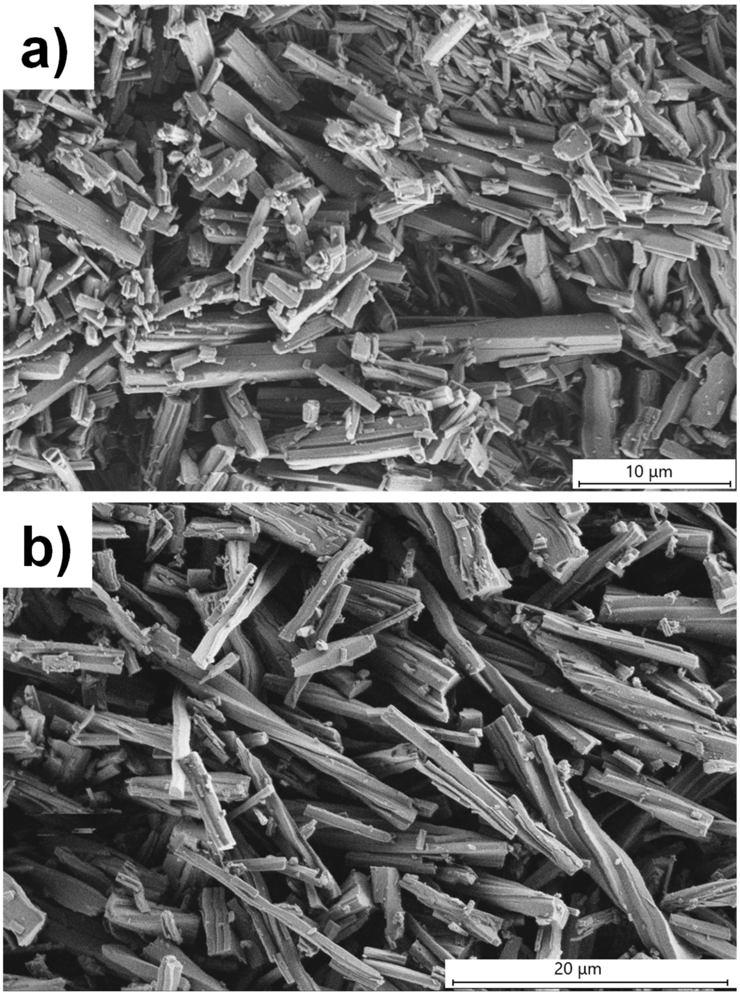

Figure S3. SEM images of (a) $\mathbf{L_1}$ and (b) $\mathbf{L_3}$ xerogels in water at 1.8 wt%.

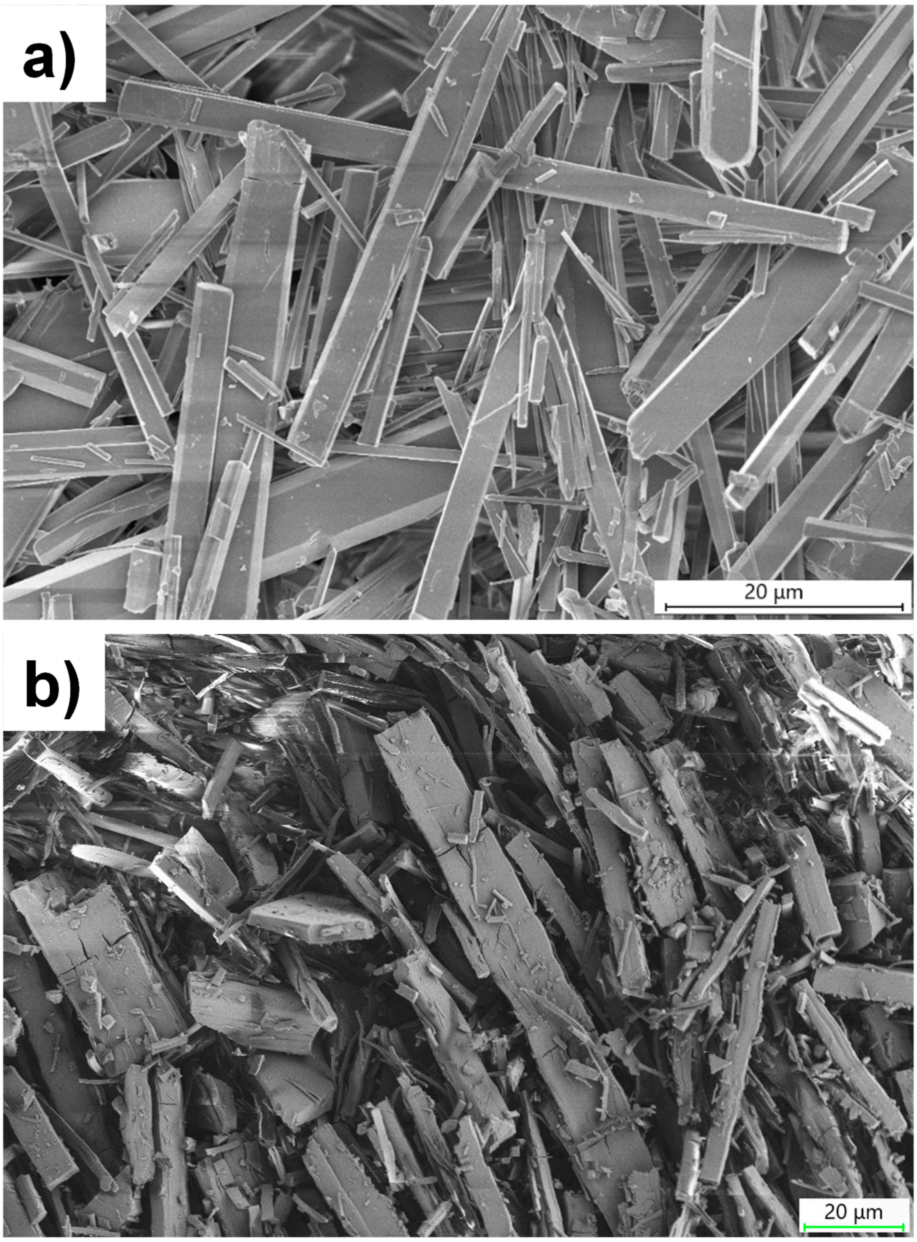

Figure S4. SEM images of the xerogels of (a) $\mathbf{L_3}$ and (b) **diNO** gels obtained from water at 4.0 wt%.

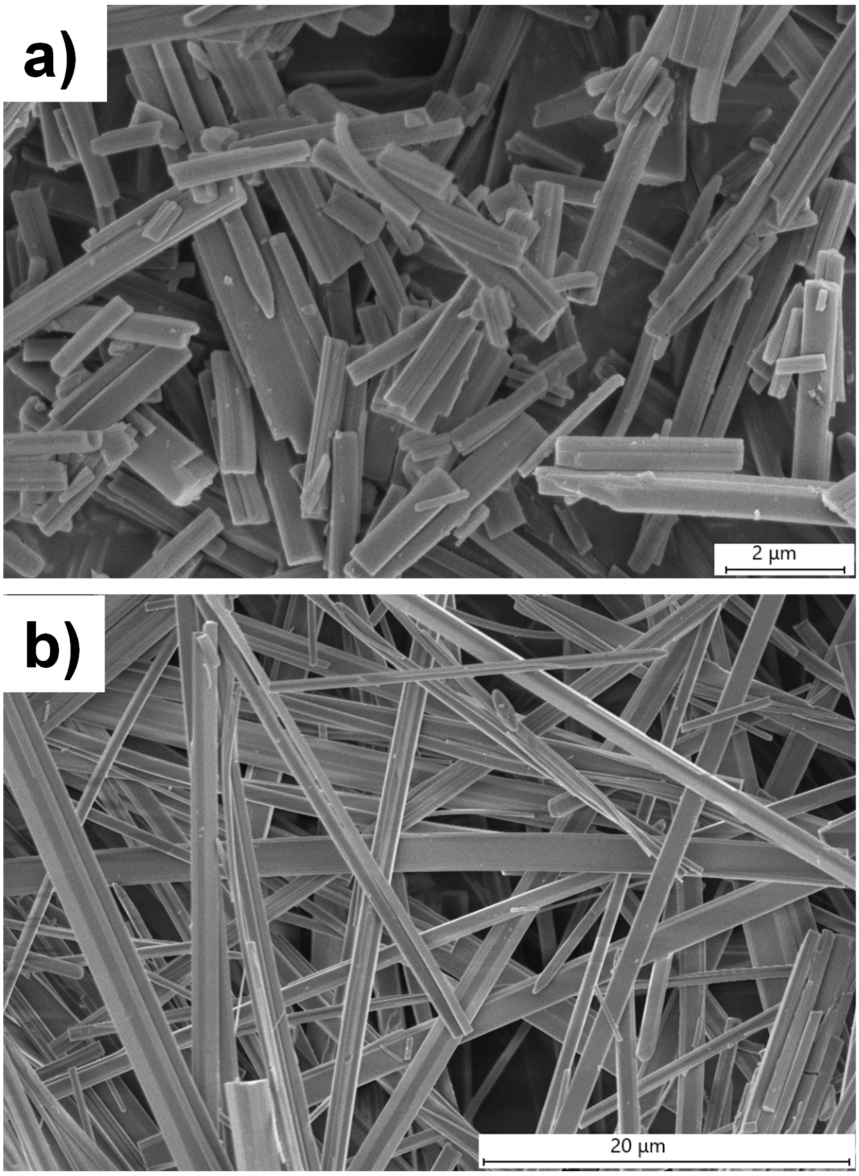

Figure S5. SEM images of $\mathbf{L_1}$ xerogels in (a) DMSO/water (1:1, v/v) and (b) methanol/water (1:1, *v*/*v*) at 2.0 wt/v%.

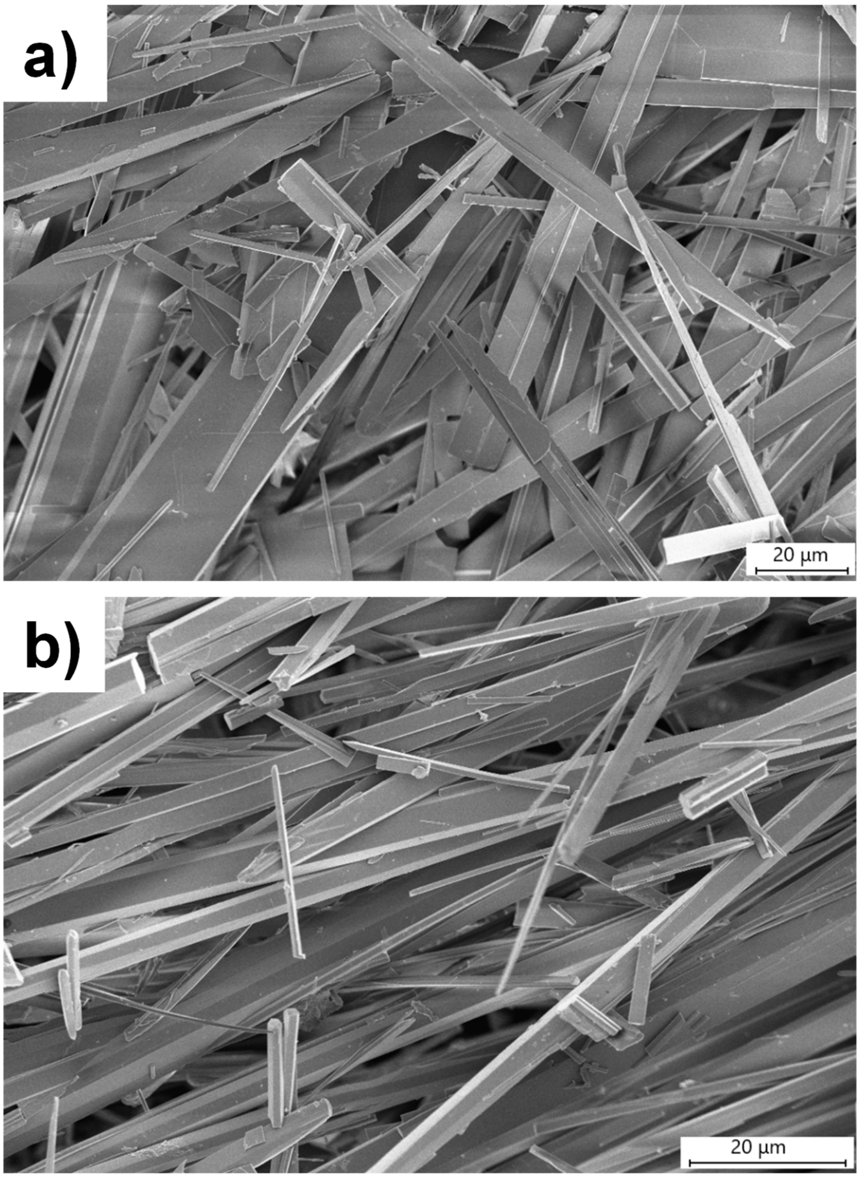

Figure S6. SEM images of $\mathbf{L_3}$ xerogels from (a) DMSO/water (1:1, *v*/*v*), and (b) methanol/water (1:1, *v*/*v*) at 2.0 wt/v%.

4. X-ray crystallography

Table S1: Crystal data

**Crystal data**	**L$_3$**•H_2O	**L$_3$**•$2H_2O$
Empirical formula	$C_{11}H_{11}N_3O_4$	$C_{11}H_{11}N_3O_4$
Color	Colorless	Colorless
Formula weight	249.23	249.23
Crystal size (mm)	0.19 x 0.05 x 0.03	0.22 x 0.16 x 0.10
Crystal system	Monoclinic	Monoclinic
Space group	$P2_1/c$	$P2/c$
a (Å)	3.73500(10)	8.1758(8)
b (Å)	12.7975(4)	5.6771(5)
c (Å)	22.9071(8)	23.529(2)
α (°)	90	90
β (°)	90.0100(14)	97.127(2)
γ (°)	90	90
Volume (Å^3)	1094.93(6)	1083.66(18)
Z	4	4
$D_{calc.}$(g/cm^3)	1.512	1.528
F(000)	520	520
μ (mm^{-1})	0.998 (CuK$_\alpha$)	0.119 (MoK$_\alpha$)
Temperature (K)	302(2)	296(2)
Reflections collected/ unique/observed [I>2σ(I)]	15306/ 1730/ 1369	17613/ 2500/ 2071
Data/restraints/parameters	1730/0/171	2500/0/172
Goodness of fit on F^2	1.045	1.062
Final R indices [I>2σ(I)]	R_1 = 0.0457<br>wR_2 = 0.1126	R_1 = 0.0409<br>wR_2 = 0.1062
R indices (all data)	R_1 = 0.0624<br>wR_2 = 0.1227	R_1 = 0.0524<br>wR_2 = 0.1144

Table S2: Hydrogen bonding parameters

**Compound L_3•H_2O**						
No.	Donor—H···Acceptor	D–H(Å)	H···A(Å)	D···A(Å)	∠D—H···A(°)	Symmetry operation
1	O(18)—H(1)···O(15)	1.00(3)	1.79(3)	2.782(3)	170(3)	1+x,y,z
2	O(18)—H(2)···O(15)	0.97(5)	1.85(5)	2.796(3)	165(4)	x,y,z
3	O(18)—H(2)···N(14)	0.97(5)	2.48(4)	3.318(3)	145(4)'	x,y,z
4	N(10)—H(10)···O(1)	0.86	2.01	2.852(2)	166	1-x,2-y,-z
5	C(4)—H(4)···O(15)	0.93	2.58	3.266(3)	131	1+x,3/2-y,-1/2+z
6	C(7)—H(7)···O(1)	0.93	2.47	3.354(3)	158	-x,2-y,-z
7	C(13)—H(13)···O(9)	0.93	2.52	3.166(3)	127	-x,1-y,-z
8	C(16)—H(16)···O(18)	0.93	2.55	3.235(3)	131	1-x,1/2+y,1/2-z
**Compound L_3•$2H_2O$**						
No.	Donor—H···Acceptor	D–H(Å)	H···A(Å)	D···A(Å)	∠D—H···A(°)	Symmetry operation
1	N(10)—H(10)···O(1)	0.86	2.07	2.8956(16)	161	1-x,-y,1-z
2	O(18)—H(18)···O(17)	0.87(2)	1.88(2)	2.7447(18)	175(2)	1-x,2-y,1-z
3	O(19)—H(19)···O(17)	0.91(2)	1.91(2)	2.8185(19)	173(2)	x,2-y,-1/2+z
4	C(7)—H(7)···O(1)	0.93	2.35	3.2486(18)	163	1-x,-y,1-z
5	C(13)—H(13)···O(9)	0.93	2.37	3.1134(17)	137	-x,2-y,1-z
6	C(15)—H(15)···O(18)	0.93	2.39	3.129(2)	136	1-x,1-y,1-z
7	C(16)—H(16)···O(1)	0.93	2.39	3.155(2)	139	1-x,-y,1-z

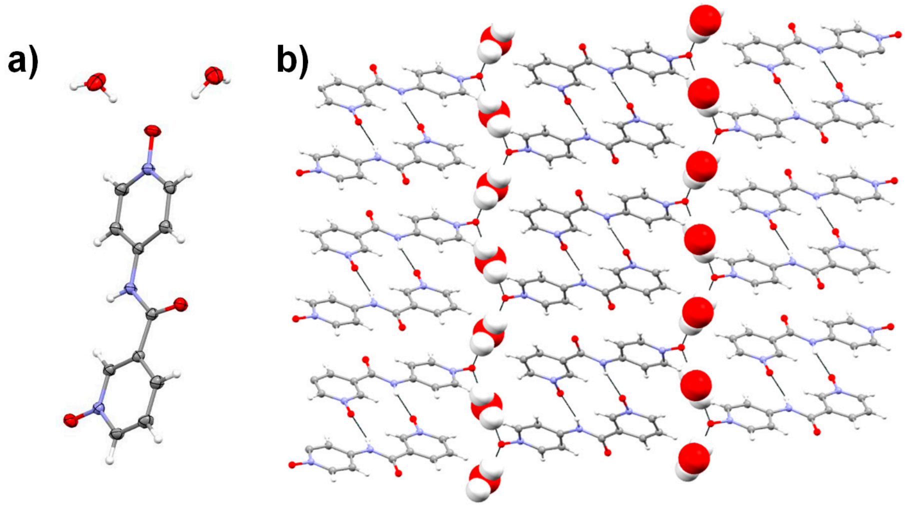

Figure S7. (a) Molecular structure of $\mathbf{L_3}$•$2H_2O$ and (b) two-dimensional hydrogen-bonded network with water molecules (space fill model) located in the cavity.

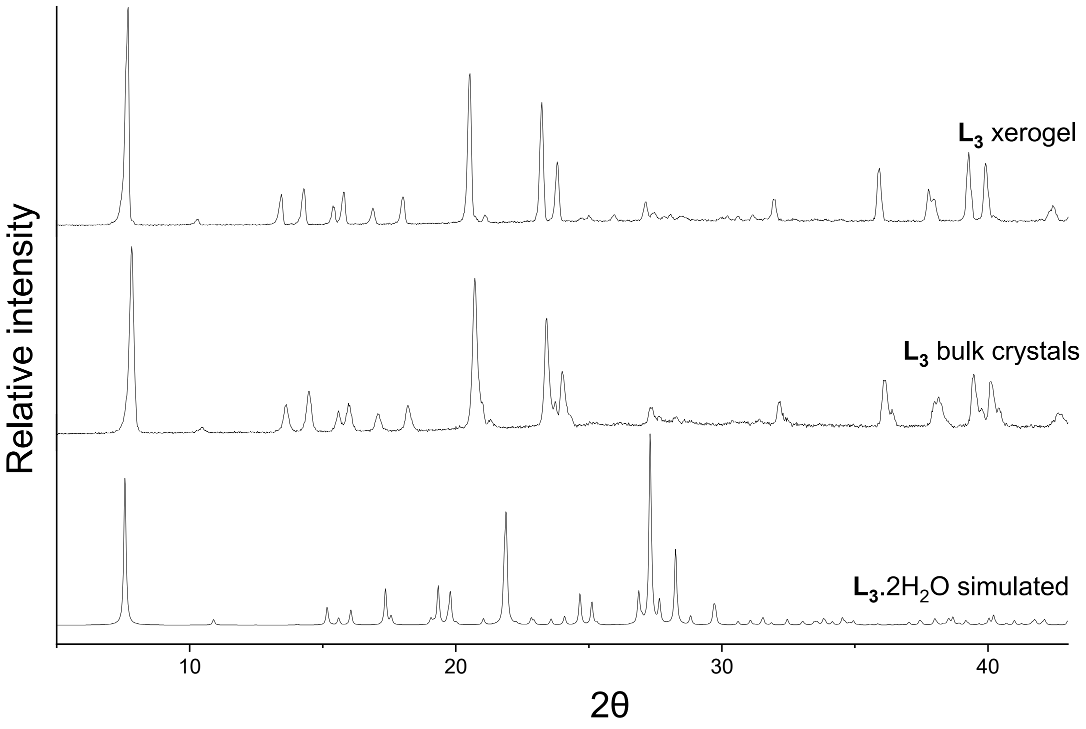

Figure S8. Comparison of the simulated pattern of the single-crystal X-ray structure of $\mathbf{L_3}$.$2H_2O$ with the PXRD pattern of the bulk crystals obtained from water, and xerogel from water at 2.0 wt%.

5. Physical properties of the gels in the presence of salts

Table S3: Stimuli-responsive properties of the gelators $\mathbf{L_1}$ and $\mathbf{L_3}$: Anion sensing in water at 1.5 wt%

Salts (1.0 equiv.)	L_1	L_3
NaF	G	G
NaCl	G	G
NaBr	G	G
NaI	Ppt	PG
KF	G	G
KCl	G	G
KBr	G	G
KI	G	G
$NaNO_3$	C	G
$NaSO_4$	C	Ppt
NaN_3	C	G
KCN	C	S
$KClO_4$	Ppt	PG
KPF_6	C	G
KBF_4	C	G
KSCN	C	PG
$K_2C_2O_4$	C	G
KNO_3	C	G

C-colloid, G-gel, PG-partial gel, Ppt-Precipitate

Table S4: Increase in G' values of the gelators at 1.5 wt% in the presence of various sodium and potassium salts in comparison with the hydrogels (1.8 wt%)

Salt added	Equivalence (equiv.)	L_1 1.5%	L_3 1.5%
NaF	1.0	2.0-fold	2.8-fold
KF	1.0	2.6-fold	3.0-fold
NaCl	1.0	1.8-fold	4.3-fold
KCl	1.0	3.0-fold	5.7-fold
NaBr	1.0	1.5-fold	3.0-fold
KBr	1.0	3.0-fold	2.3-fold
KI	1.0	---	1.7-fold
KNO_3	1.0	---	1.9-fold
KBF_4	1.0	---	4.8-fold
KPF_6	1.0	---	5.0-fold
$K_2C_2O_4$	1.0	---	1.8-fold

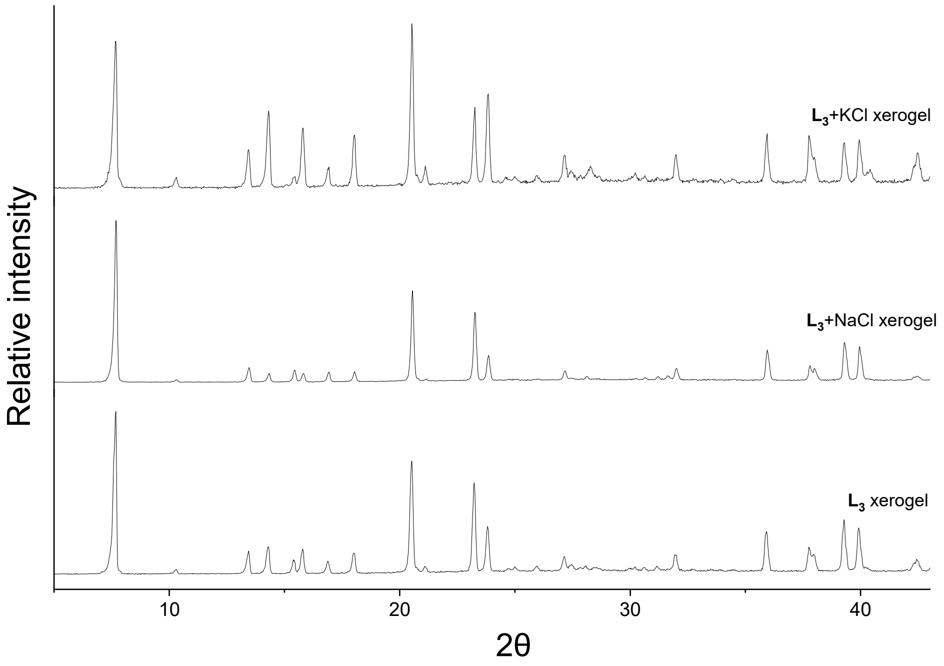

Figure S9. PXRD pattern of xerogels obtained from the hydrogel of L_3 at 2.0 wt% and in the presence of 1.0 equivalence of NaCl and KCl.

Table S5: Stimuli-responsive properties of the gelators L_1 and L_3: Cation sensing in water at 1.5 wt%

Salts (1.0 equiv.)	L_1	L_3
CsCl	G	G
$MgCl_2$	G	G
$CaCl_2$	G	G
$SrCl_2$	G	G
$BaCl_2$	G	G
$AlCl_3$	S	PG
NH_4Cl	PG	G
$CuCl_2$	Ppt	PG
$ZnCl_2$	Ppt	G
$CdCl_2$	Ppt	G

Table S6: Increase in G' values of the gelators at 1.5 wt% in the presence of chloride salts of various cations in comparison with the hydrogels (1.8 wt%)

Salt added	Equivalence	L_1	L_3
CsCl	1.0	1.1-fold	3.8-fold
$MgCl_2$	1.0	1.9-fold	4.8-fold
$CaCl_2$	1.0	1.7-fold	5.0-fold
$SrCl_2$	1.0	1.8-fold	2.3-fold
$BaCl_2$	1.0	1.5-fold	1.8-fold
NH_4Cl	1.0	---	1.6-fold
$CuCl_2$	1.0	---	-----
$ZnCl_2$	1.0	---	2.8-fold
$CdCl_2$	1.0	---	24.7-fold

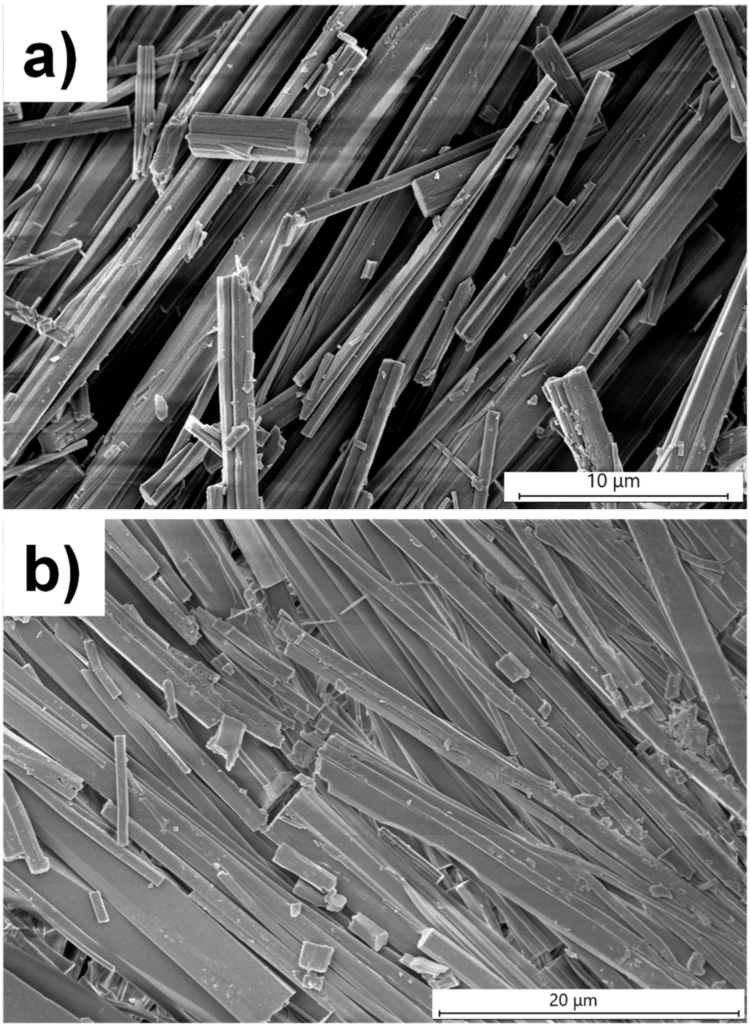

Figure S10. SEM images of xerogels of (a) $\mathbf{L_1}$ and (b) $\mathbf{L_3}$ at 2.0 wt% obtained from water in the presence of 1.0 equivalence of $MgCl_2$.

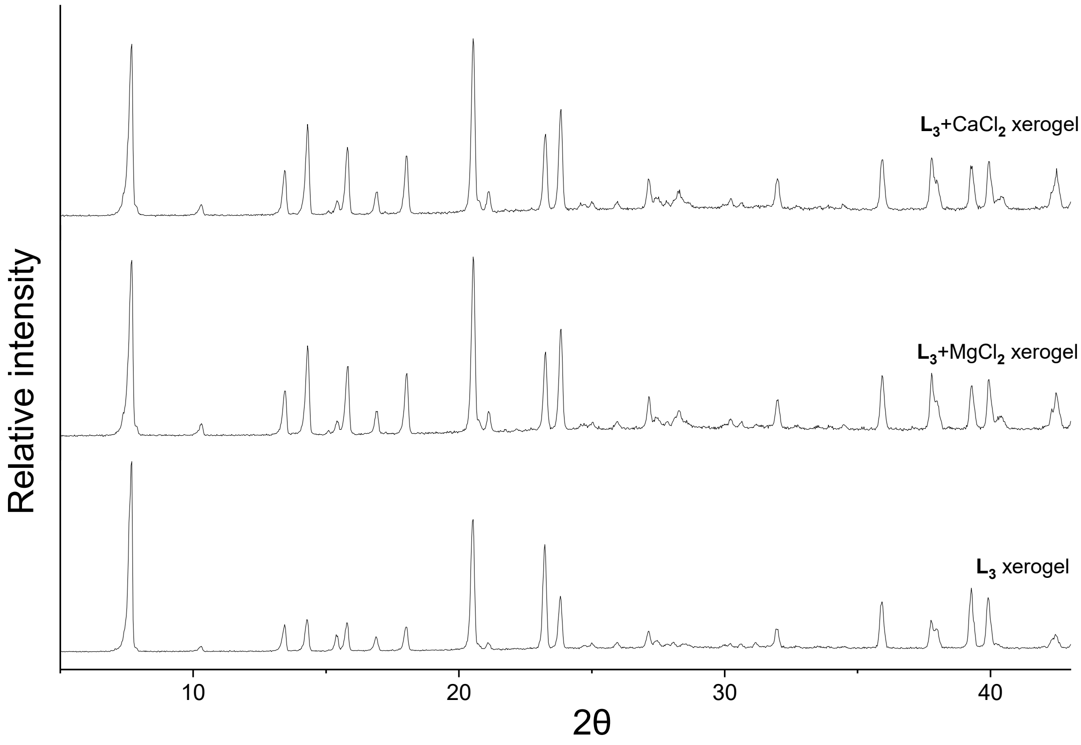

Figure S11. Comparison of the PXRD pattern of xerogels (2.0 wt%) of **L_3** hydrogels, and the gels in the presence of 1.0 equivalence of $MgCl_2$ and $CaCl_2$.

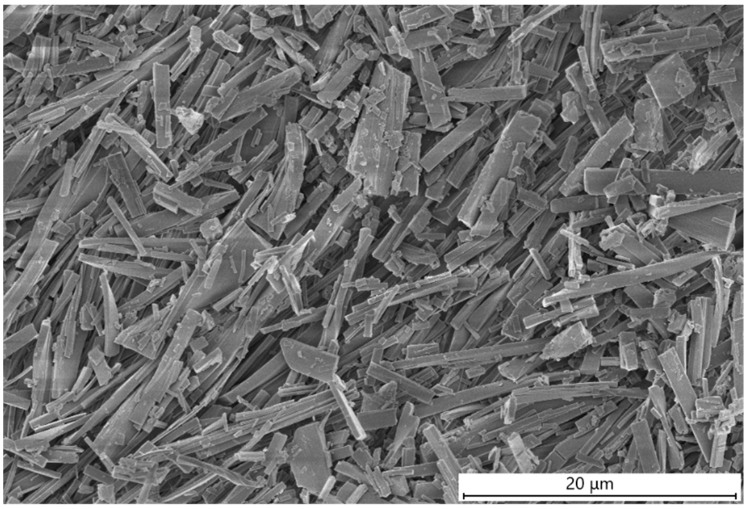

Figure S12. SEM images of the xerogels of **L_3** in the presence of 1.0 equivalence of $CdCl_2$ in water at 2.0 wt%.

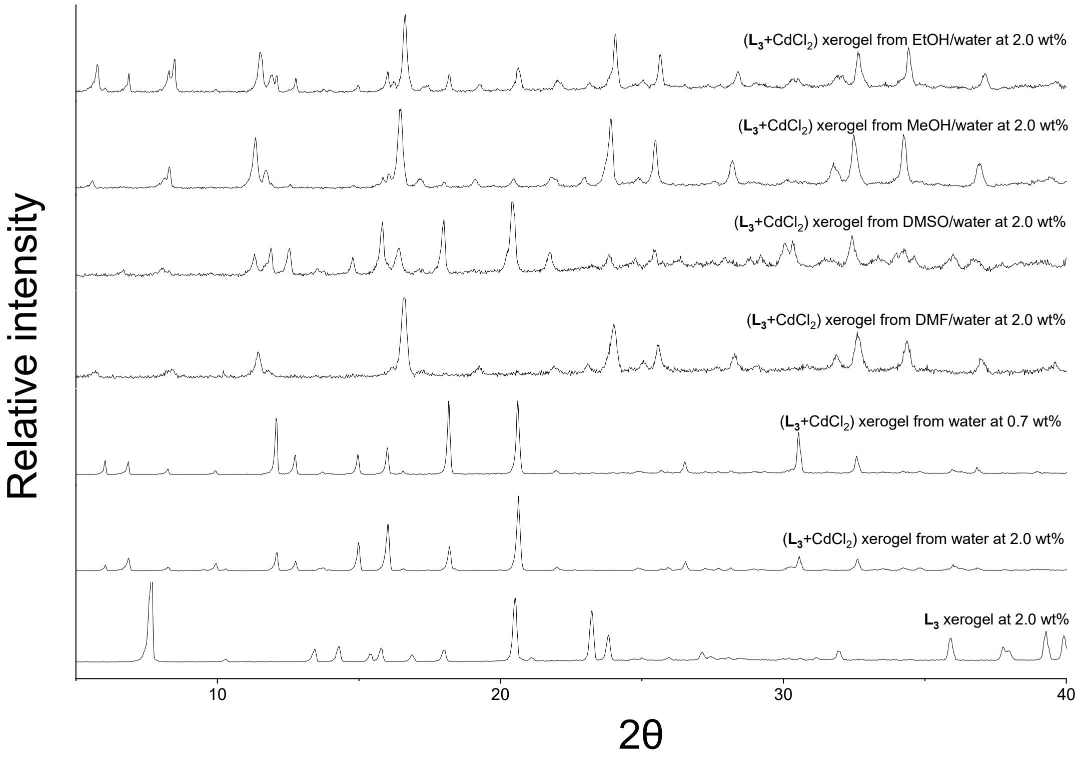

Figure S13. Comparison of the PXRD pattern of **L₃** xerogel with the PXRD pattern of the xerogels of **L₃**-$CdCl_2$ mixture in various solvents.

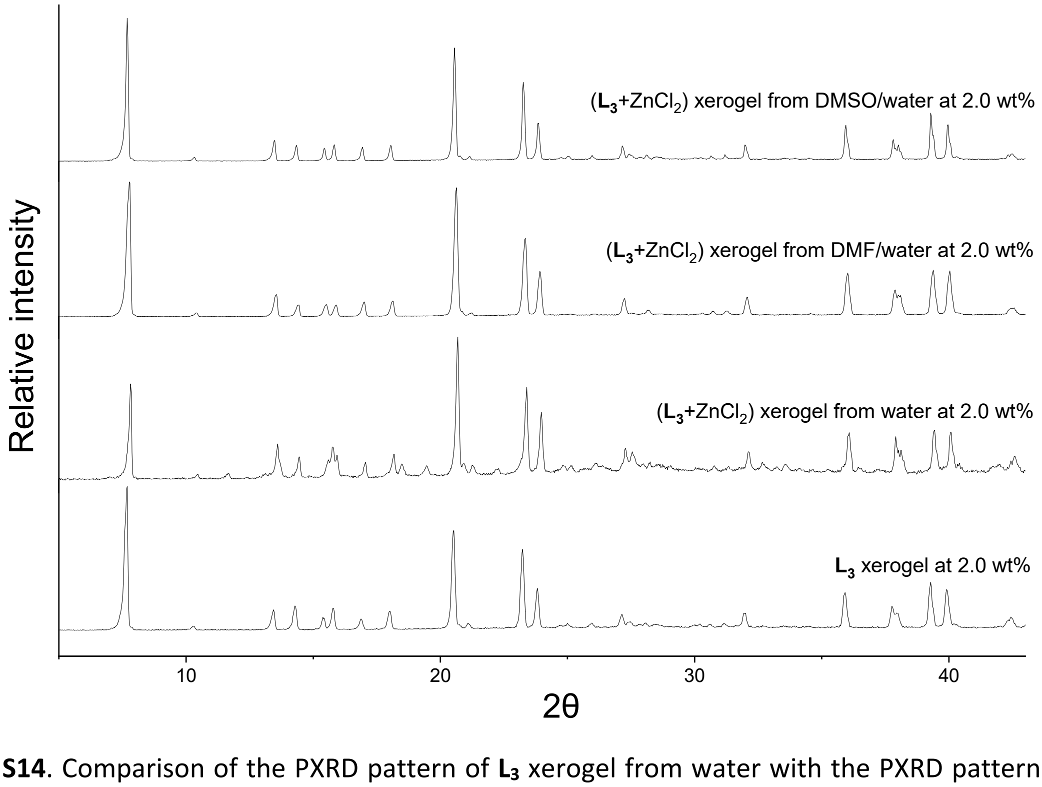

Figure S14. Comparison of the PXRD pattern of **L₃** xerogel from water with the PXRD pattern of the xerogels of the mixture (**L₃** + $ZnCl_2$) in various solvents.

6. NMR spectra

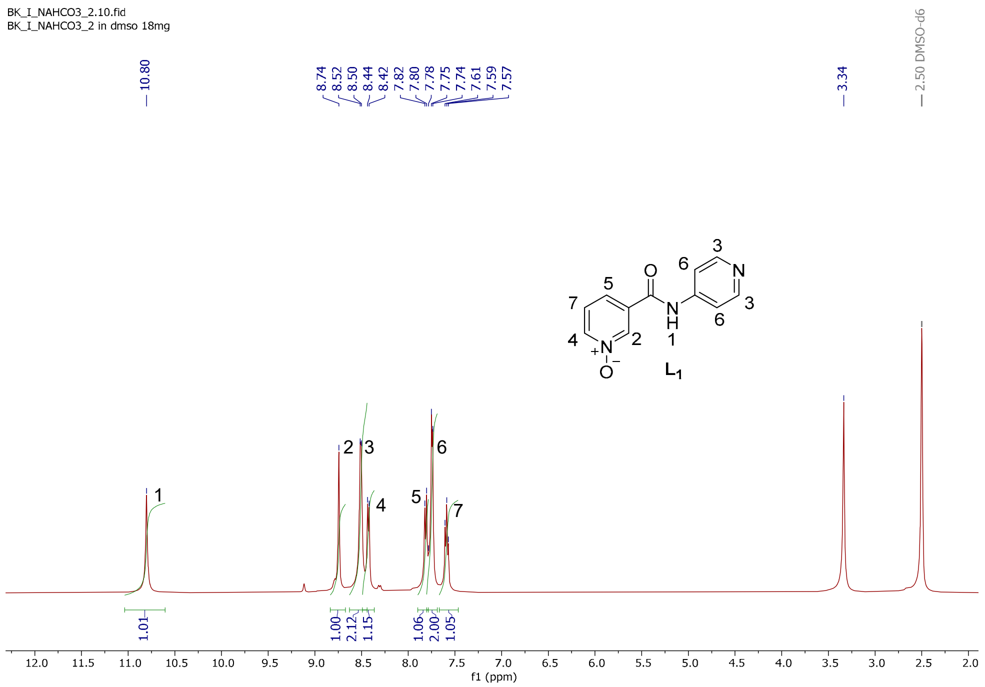

Figure S15. ^{1}H NMR spectrum of compound **L_1**.

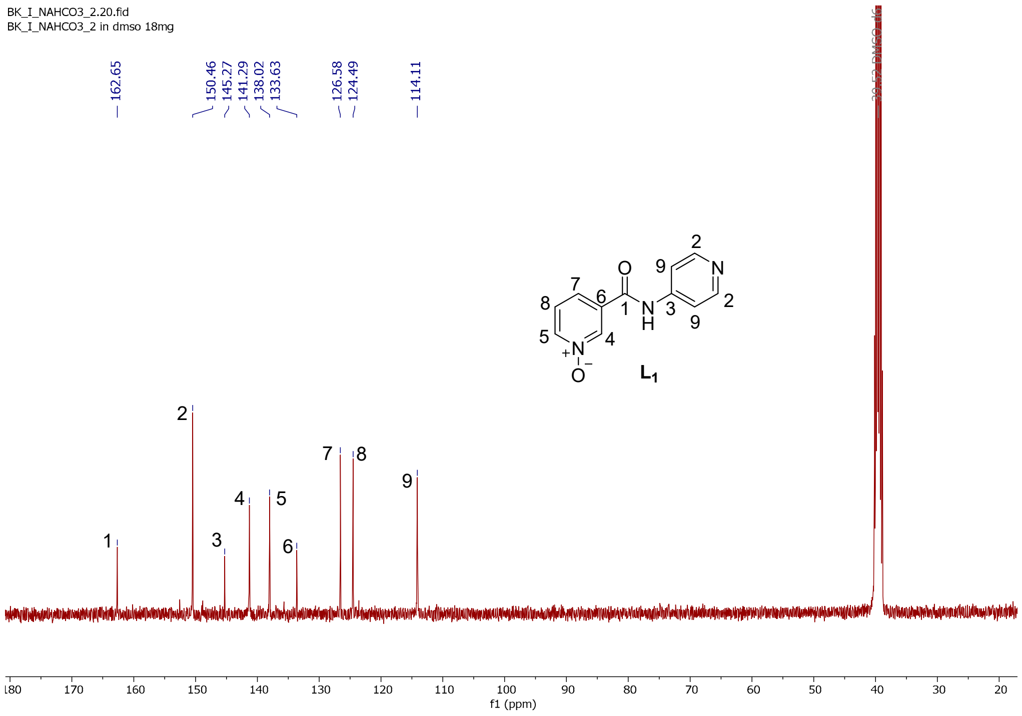

Figure S16. ^{13}C NMR spectrum of compound **L_1**.

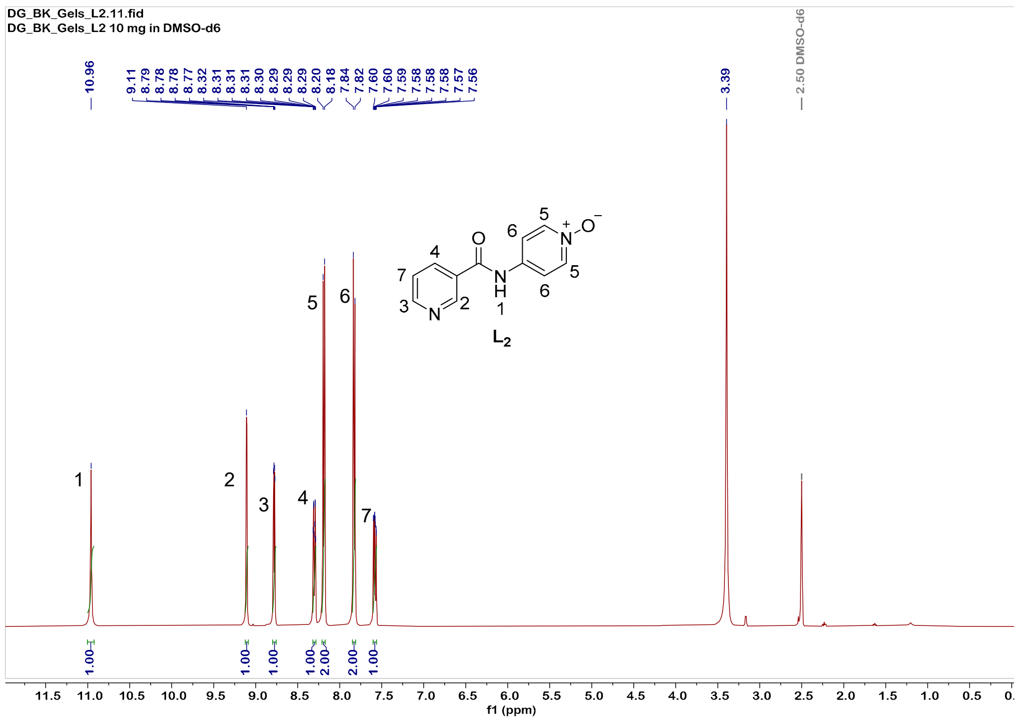

Figure S17. ^{1}H NMR spectrum of compound $\mathbf{L_2}$.

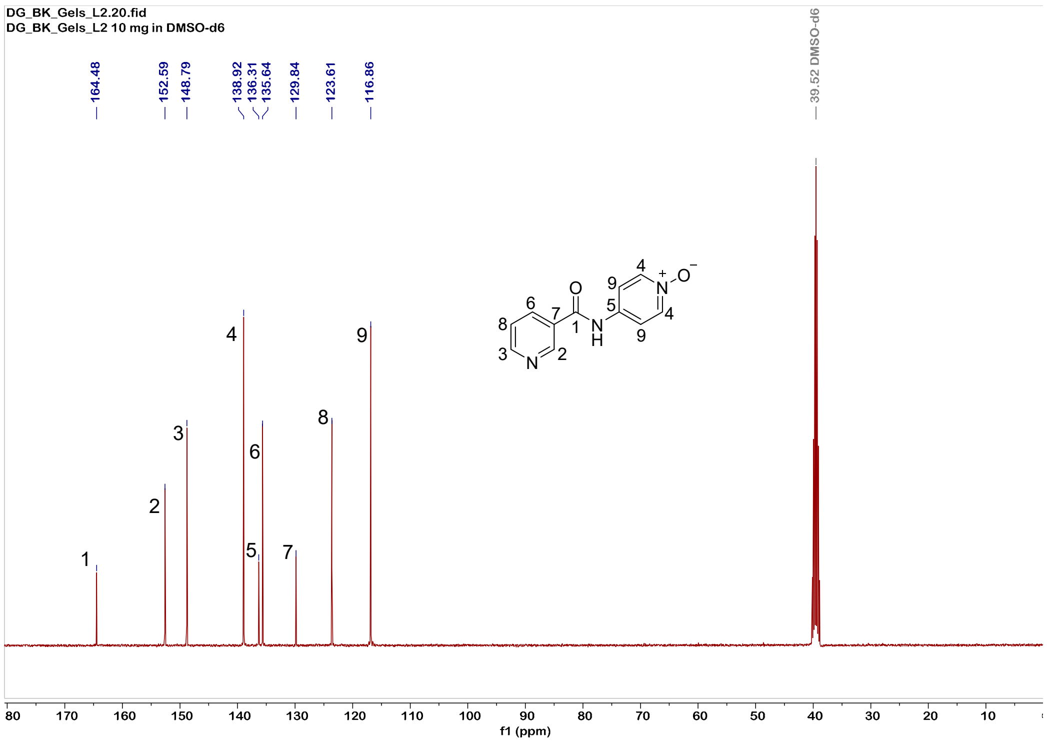

Figure S18. ^{13}C NMR spectrum of compound $\mathbf{L_2}$.

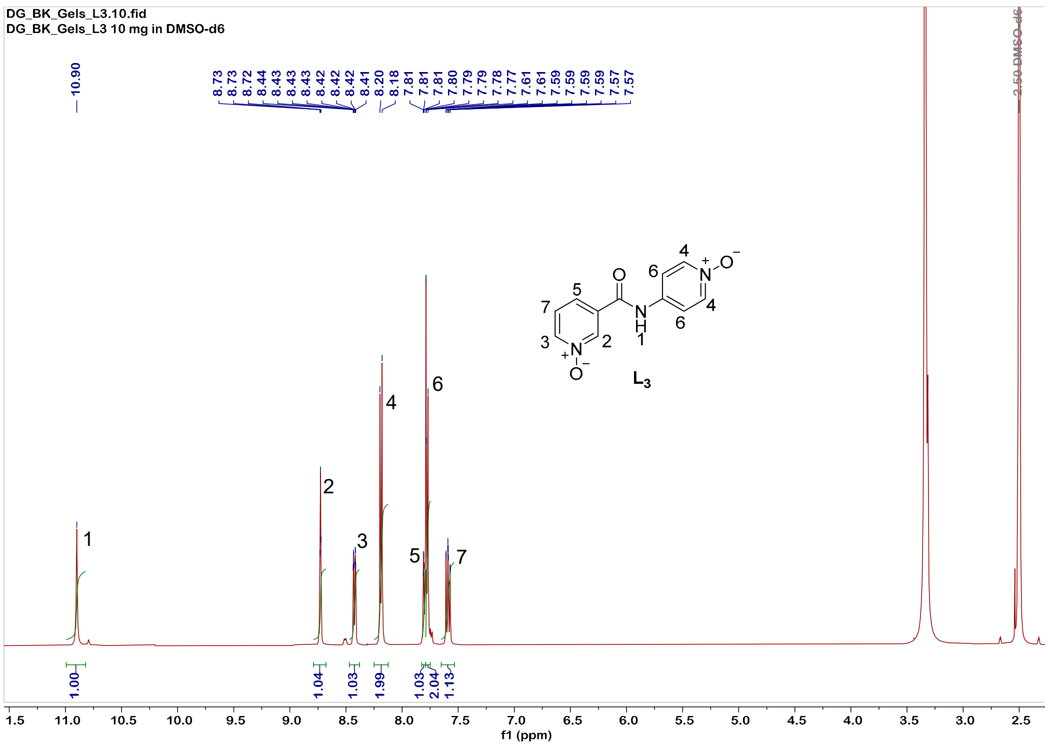

Figure S19. ^{1}H NMR spectrum of compound $\mathbf{L_3}$.

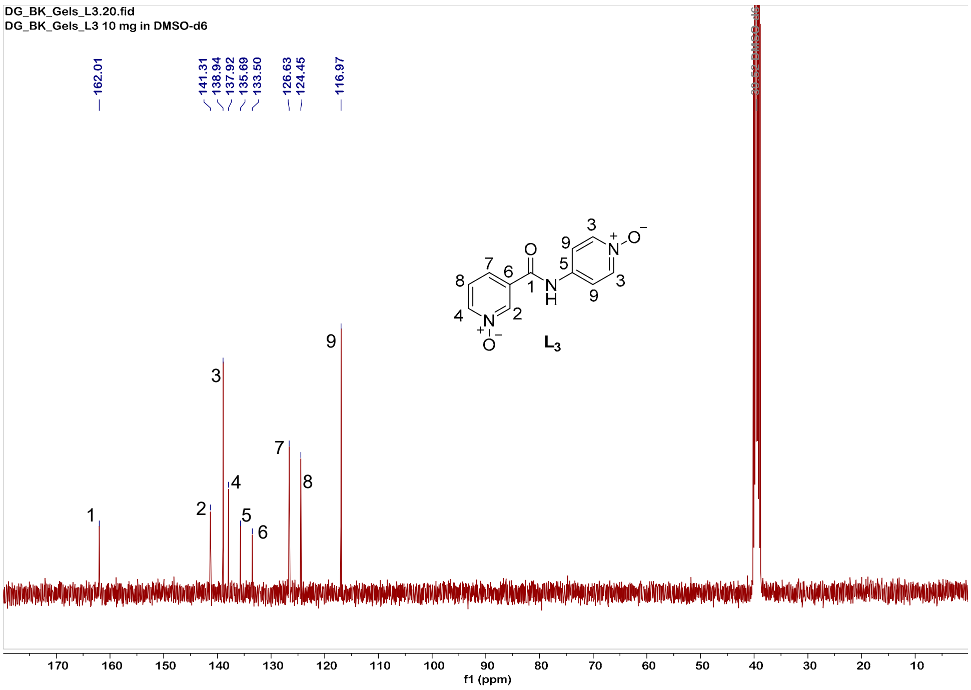

Figure S20. ^{13}C NMR spectrum of compound $\mathbf{L_3}$.
